# Dynamic and programmable morphology and size evolution via a living hierarchical self-assembly strategy

**DOI:** 10.1038/s41467-018-05142-3

**Published:** 2018-07-17

**Authors:** Xing Wang, Peiyuan Gao, Yanyu Yang, Hongxia Guo, Decheng Wu

**Affiliations:** 1 Beijing National Laboratory for Molecular Sciences, State Key Laboratory of Polymer Physics & Chemistry, Institute of Chemistry, Chinese Academy of Sciences, Beijing, 100190 China; 2 Pacific Northwest National Laboratory, Richland, 99352 WA USA; 3 College of Materials Science and Engineering, Zhengzhou University, Zhengzhou, Henan 450001 China; 4 University of Chinese Academy of Sciences, Beijing, 100049 China

## Abstract

Recent advances in the preparation of shape-shifting and size-growing nanostructures are hot topics in development of nanoscience, because many intelligent functions are always relied on their shape and dimension. Here we report a tunable manipulation of sequential self-assembled transformation in situ via a hierarchical assembly strategy based on a living thiol–disulfide exchange reaction. By tailoring the external stimuli, the reactive points can be generated at the ends of initially unimolecular micelles, which subsequently drive the pre-assemblies to periodically proceed into the hierarchically micellar connection, axial growth, bending, and cyclization processes from nanoscopic assemblies to macroscopic particles. Of particular interest would be systems that acquired the shape control and size adjustment of self-assemblies after termination or reactivation of disulfide reshuffling reaction by regulating external stimuli whenever needed. Such a hierarchical strategy for self-assembled evolution is universally applicable not only for other disulfide-linked dendritic polymers but also for exploitation of biological applications.

## Abstract (web summary)

Controlling shape-shift and size-growth in nanostructures are important developments in nanoscience but controlling morphology change with an instant on/off function remains challenging. Here the authors demonstrate control over morphology and size transformation of POSS molecules based on living thiol-disulfide exchange reactions.

## Introduction

The “bottom up” or “engineering up” approach from a single molecule to functional architectures has a great significance in understanding biologically spontaneous self-assembly process, which offers an efficient strategy for creating a myriad of well-organized nanostructures in applications of drug delivery, cosmetics, dispersant technology, photonic detector, sensor, and nanoreactor arenas^1–12^. Recently, a great effort has been focused on the dynamic process of solution-assembled nanostructures in highly selective solvents through the elaborate design of the molecular architectures and careful control of the external environments to study the real-time self-assembled evolution. The preparation of shape-shifting and size-growing structures is emerging as one of the advanced strategies in organic-based nanoscience and nanotechnology, because most of the smart functionalities of nanocarriers are directly determined by their shapes and sizes, which not only provides detailed information on the structural transformation but also guides the outcome of morphology and functionality in response to outside stimuli. Frequently used methodologies to create temporal evolution of the self-assembly pathway always require precise polymer syntheses, sophisticated architectural design (hydrophobic/hydrophilic balance, composition, geometry, dimension, surface chemistry, and flexibility)^13–35^, fine control of the environmental conditions (pH, temperature, stress, photons, ultrasound, ionic strength, and solvents) and sophisticated optimization of solution assembly kinetic pathways^36–45^. Nevertheless, these methods are still unable to delicately manipulate the self-assembly system and veritably obtain the full-scale solution assemblies due to the difficulty in achieving the precise control of amphiphilic inherent essences at any moment once the self-assembly system is initiated. In other words, regulation of real-time morphology evolution with an instant on/off function at arbitrary time to gain the desired self-assemblies remains a grand challenge. To circumvent this problem, it is greatly desired to design the manipulative reactions and conditions to tailor self-assembly systems with the strict criteria that the self-assembly behaviors should be able to be activated, controllably terminated and interrupted, and reinitiated by the external stimuli whenever needed, because many envisioned applications endeavor to exploit solution assemblies as intelligent nanocontainers for the high encapsulation and controlled release of small molecule cargoes, requiring a detailed understanding of the dynamic processes, long-term stability, and stimulative responsibility of solution assemblies.

Our previous studies exploited a living controlled hydrogel formation method for tailor-made in situ gelling systems^46,47^. Specifically, a star-shaped POSS-(SS-PEG)_8_ molecule (Supplementary Fig. 1), containing a disulfide-linked core/shell structure with one polyhedral oligomeric silsesquioxane (POSS) core and eight easy-leaving polyethylene glycol (PEG) shells, was adopted to produce various hybrid hydrogels with customized structures and properties based on a pH-responsive on/off reaction. Supplementary Fig. 2 illustrated the mechanism of pH-triggered thiol–disulfide exchange reaction that could result in the dissociation of PEG shells and linkage of POSS cores. The driving force was shifting of equilibrium toward the stable disulfide-linked products through the release of PEG shells on account of the selective core/shell separations. So, rational control of system pH and reaction time can tailor the hydrophobic/hydrophilic ratios and topologies, thus manipulating the self-assembled architectures and dimensions in aqueous media. In this case, it is believed that the continuously time-dependent morphology transformation and size evolution with an instant on/off function can be facilely achieved by this switchable method. Of particular interest would be systems that acquired the shape control and size adjustment of self-assemblies after termination or reactivation of the disulfide reshuffling reaction by regulating the system pH.

**Fig. 1:**
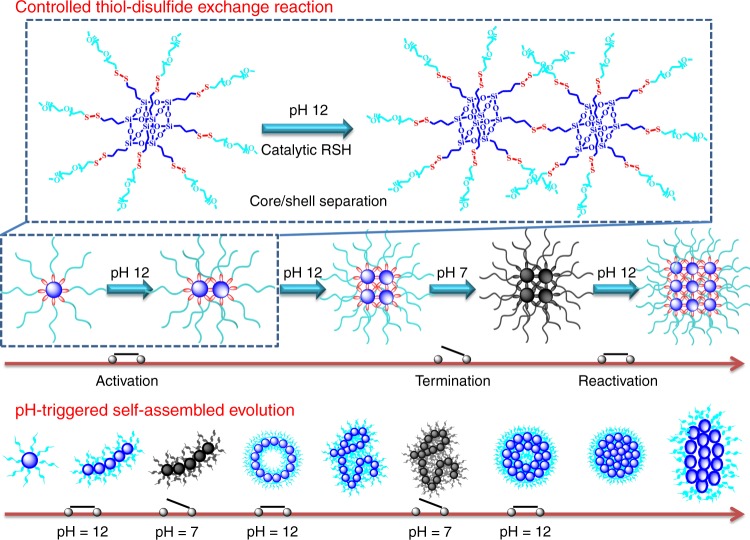
Controllable strategy for morphology evolution with a pH-switched on/off function. Schematic representation of the living controlled thiol–disulfide exchange reaction and the pH-triggered self-assembled evolution. When the thiol–disulfide exchange reaction is activated in the presence of catalytic thiols in pH 12 solutions, the selective core/shell separation gradually leads to the continuous connections of POSS-embedded cores; in this case, the self-assembled evolution is performed from unimolecular micelles to the elliptic nanoparticles. Once neutralizing the system pH to 7 at any moment, the exchange reaction is deactivated, and thus no further morphology evolution is conducted any more. However, these stable inert sites can be reactivated by basification whenever needed, and the trapped morphology evolution can return back on the previous track

Herein we propose a living hierarchical self-assembly strategy to manipulation of the morphology and size evolution in situ by tuning the pH stimuli to activate and/or terminate the thiol–disulfide exchange reaction of the amphiphilic POSS-(SS-PEG)_8_ polymer (Fig. 1). By activing the disulfide reshuffling reaction in aqueous solutions, the constructive linkage of POSS-embedded domains and removal of PEG segments caused the amphiphiles gather into unimolecular micelles, which acted as the pre-assemblies that further proceeded to the micellar connection, axial growth, bending, and cyclization processes driven by the reactive points at the ends with high-sufficient activity, presenting a periodically hierarchical self-assembly behavior on the all of nanoscales, microscales, and macroscales. Besides, the rigid POSS-embedded backbone and strong aggregation ability can furnish these POSS-containing hybrid polymers with unique self-assembly behaviors^48–57^, thereby generating various nanostructures with a second level of hierarchy. Notably, such a real-time self-assembled evolution could be interrupted at any point of time by neutralization or restarted by rebasification. Using this principle, many sophisticated materials with controlled structures and special properties may have great applications in biomedical platform.

## Results

### pH-triggered morphology and size evolution in situ

**Fig. 2:**
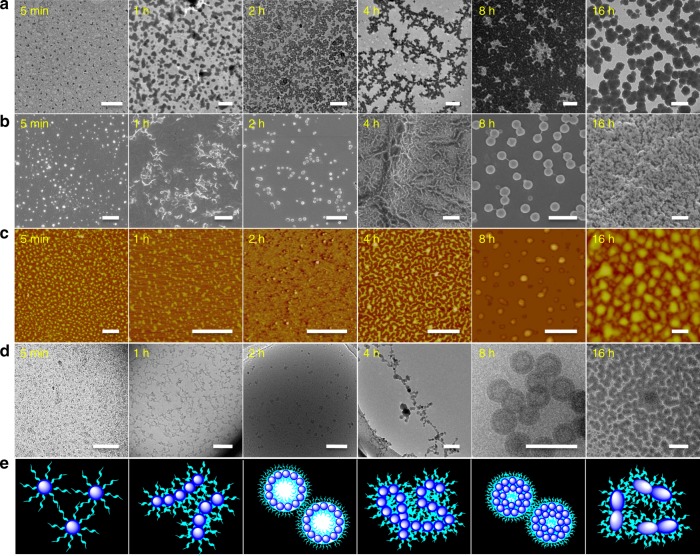
Multiple measurements of the morphology evolution. After neutralizing the aqueous solution and removing the by-products before dialysis, all the samples are prepared from the neutralized aqueous solution unless otherwise noted. **a** TEM, **b** SEM, **c** AFM, **d** Cryo-TEM, and **e** Schematic images showing the self-assembled evolution of POSS-SS-(PEG)_8_ with the concentration of 1 mg/mL in pH 12 aqueous solutions for various times. Scale bars: 0.5 μm. Along with the initiation of morphology transition in alkaline solutions, multifarious morphologies like uniform micelles, cylinders, vesicles, worm-like micelles, hollow spheres, and elliptic nanoparticles are generated in sequence. The blue sphere represents the hydrophobic POSS core and the cyan chain represents the PEG shell within the self-assemblies

Effective adjustment of thiol–disulfide exchange reaction kinetics is crucial for acquiring more opportunities to observe the self-assembled evolution. With the aim of reducing the exchange reaction rate and avoiding the gelation, the concentration of POSS-(SS-PEG)_8_ polymer was set as 1 mg/mL in pH 12 solutions. The good hydrophilicity of PEG shells made the amphiphilic polymers disperse well and keep stable after basification for 5 min, forming the unimolecular micelles with the diameter of ~5 nm (TEM) and 3 nm by all-atom molecular dynamics (MD) simulation (Supplementary Fig. 3). After addition of catalytic amounts of cysteamine, exchange reaction was immediately activated along with the initiate of PEG chains dissociation and POSS core linkage. With a greater extent of exchange, more PEG segments were released and a higher degree of crosslinking reaction was achieved. Accordingly, the hydrophobic/hydrophilic ratio was continuously increased along with the ongoing variation of dendritic topology. Under this circumstance, the solution assemblies in situ transformed from unimolecular micelles to spherical micelles to cylinders to vesicles to worm-like micelles then to hollow spheres and finally to elliptic nanoparticles (Fig. 2). No doubt that the cleavage and recombination of disulfides played important roles in guiding this self-assembled evolution. For this morphology transition, two types of self-assembly mechanisms may be considered and interpreted. One possibility is the continuously morphological formation, destruction and reconstruction processes due to the variation of molecular topology and amphiphilic ratio. However, the disulfide exchange reaction rate was faster than the self-organization process in alkaline environment. In this case, it was believed that the main driving force of morphology transition should be relied on the hierarchical self-assembly behaviors, by which the thiol–disulfide exchange reaction occurred on the surface or interior of previous pre-assembled subunits. So many intricate morphologies were fabricated with a second level of hierarchy, achieving the periodically one-dimensional axial growth, two-dimensional cyclization, and internal crosslinking in the whole evolution process.

### Investigation into micelle growth mechanism

**Fig. 3:**
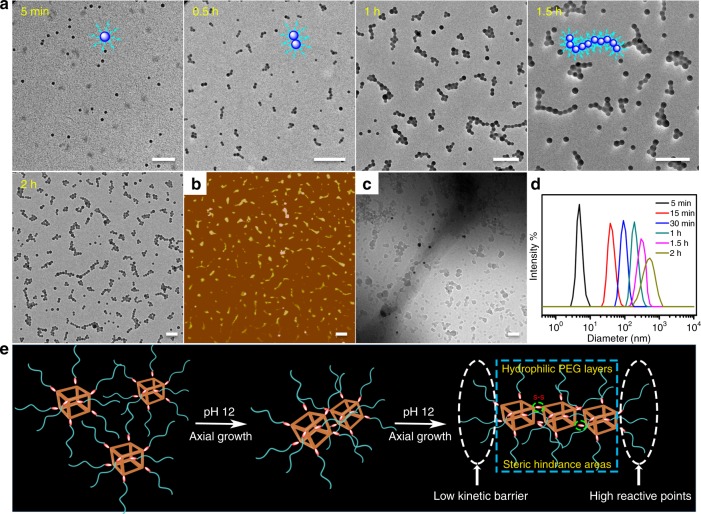
Mechanism of the hierarchically axial growth of the micelles. After neutralizing the aqueous solution and removing the by-products before dialysis, all the samples are prepared from the neutralized aqueous solution unless otherwise noted. **a** TEM images showing the self-assembled evolution of POSS-SS-(PEG)_8_ with the concentration of 1 mg/mL in pH 8 solutions for various times. Insets are the correspondingly schematic structures. **b** AFM and **c** Cryo-TEM images of the short cylindrical formations at 2 h. Scale bars: 50 nm. **d** DLS results of the nanoparticles for various times. **e** The possible self-assembled mechanism of nanostructural axial growth. In this process, the thiol–disulfide exchange reaction makes the disperse unimolecules quickly aggregate and interlink by the disulfides, thus forming many oligomer-linked cylinders from the pre-micelles with the hierarchical self-assembly behaviors. The low kinetic barrier at both ends, due to the steric hindrance areas from POSS-embedded domains and the swollen layers from stretched PEG shells, furnishes these cylinders with higher reactive points at the micellar ends and drives the subunits to connect together. The orange cube represents the POSS core, the red ellipsoid represents the disulfide linkage and the cyan chain represents the PEG shell

In order to trace this dynamic morphology evolution and further reveal the hierarchical self-assembly mechanisms, reduction of the exchange reaction rate was an essential method by adjusting the system pH to 8. Although the thiols were little activated facilitating the exchange reaction, the structural transformation with a stepwise self-assembly behavior was still performed (Fig. 3). Careful observation found that this dynamic transition process was indeed a kind of second level of hierarchy from the originally molecular-level micelles. Distinct with the traditional hierarchical self-assembly that fully relied on the temperature changes, electrostatic attraction, crystallization, and solvent selectivity, such hierarchical evolution was analogous to the pH-triggered thiol–disulfide exchange reaction that occurred on the surface or interior of the nanoparticles. For simplification purposes, one POSS-SS-(PEG)_8_ unit can be modeled as an isolated core-shell sphere. Based on the sluggish exchange reaction in pH 8 solutions, the unimolecular micelles were slowly connected with each other and formed dimer, trimer, and multimer micellars, just like the structural orientation toward axial growth in one dimension until the formation of short cylindrical structures (Fig. 3a and Supplementary Fig. 4). Moreover, the unique capability of POSS aggregates to form layered crystals is the main driving force in the formation of ellipsoids that seemed like chain-like or lamellar structures parallel to the long axis^57^, so the oligomer micellar connection can gradually assemble into the long and narrow cylinders with low free energy and high priority. AFM and Cryo-TEM images (Fig. 3b, c) also exhibited the micelle-linked cylinders while DLS results (Fig. 3d) intuitively gave the proof of nanostructural size growth. This referential growth along the axial direction was attributed to the large kinetic barrier and hysteresis effects on the other directions originating from the obstruction of swollen liquid PEG layers, huge hindrance of rigid POSS units and the complex self-assembled pathway in aqueous mediums. Besides, the less coverage of PEG chains at both ends of the micellar aggregates endowed the reactive points at the ends with sufficient activity to induce the following self-assembly into cylinders, presenting a hierarchical stepwise micellar connection towards the axial direction (Fig. 3e).

### All-atom simulations of nanostructural axial growth

**Fig. 4:**
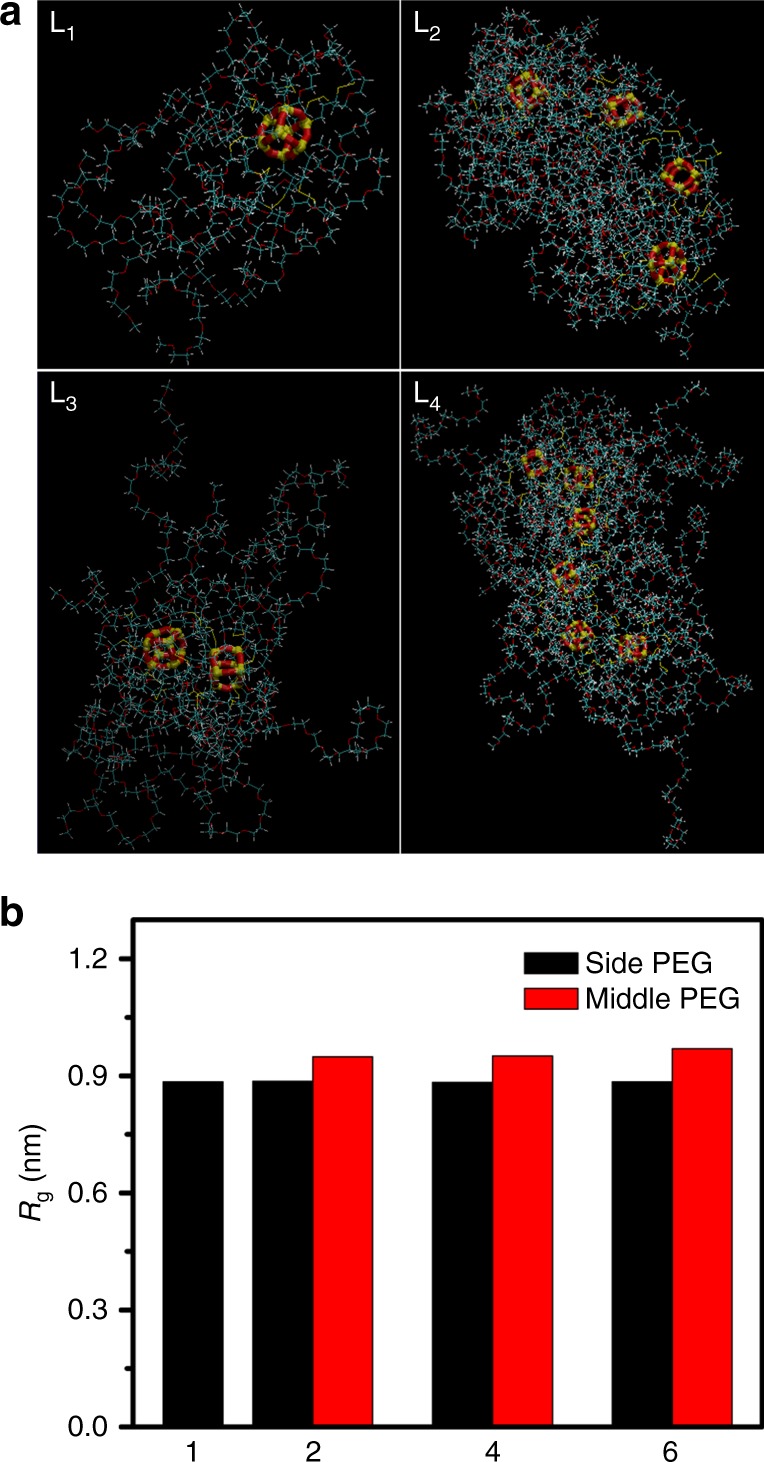
MD studies of the micellar axial growth mechanism. **a** The typical conformation of POSS_*m*_-PEG_*n*_ oligomer in water with various chain lengths at (L_1_) 1, (L_2_) 2, (L_3_) 4, and (L_4_) 6. Water molecule is not shown for clarity. The inner cube represents the POSS core, wherein the yellow cylinder represents the silicon (Si) atom and the red cylinder represents the oxygen (O) atom. The outer linkage represents the PEG shell, wherein the red cylinder represents the oxygen (O) atom, the yellow cylinder represents the sulfur (S) atom, the wathet blue cylinder represents the carbon (C) atom, and the white cylinder represents the hydrogen (H) atom. **b** The average Rg for PEG chain on the side and middle of POSS molecules (see Supplementary Table 1 for the detailed simulation parameters)

The limitation of current experimental techniques made obtaining a deep understanding of the mechanism underlying the hierarchical self-assembly difficulty. To address this challenge, we performed MD simulations to intuitively study the axial growth pathways (Supplementary Table 1). When the degree of polymerization was set as 1 and 2, the unimolecular and dimer structures were obtained. As the chain length reached more repeat units, the oligomers exhibited the cylindrical conformations (Fig. 4a). Although the joint sections between the adjacent POSS molecules were flexible, the whole chains could not bend too much due to the steric hindrance effects of side PEG chains. However, this effect also changed the conformation of the side PEG chain, especially for these PEG chains in the medium. Figure 4b showed the average root squared gyration radius (*R*_g_) of middle PEG and side PEG chains in POSS_*m*_-PEG_*n*_ oligomer. Noticed that the *R*_g_ of middle PEG chains was 10% larger than the side one, indicating the difficulty to approach the center POSS for other active micellar aggregates. So, the lower steric hindrance generated the reactive points at the both ends, driving the oligomers to connect together to form the cylinders along the axis direction with high priority.

### Nanostructural bending, cyclization, and radial connection

**Fig. 5:**
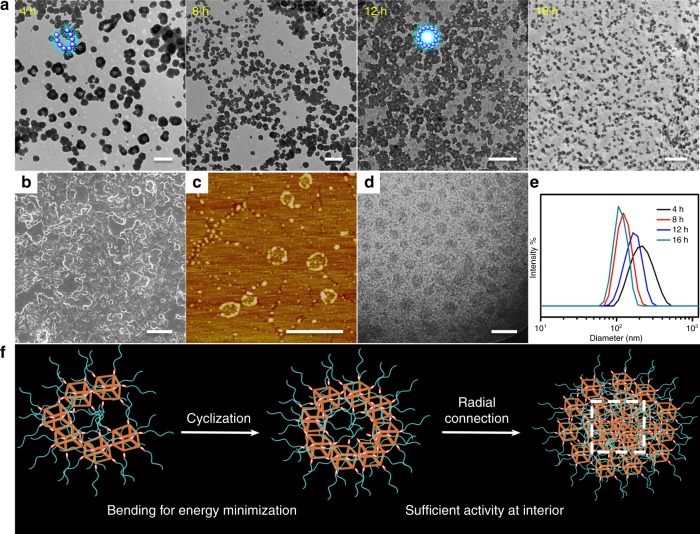
Mechanism of the hierarchical bending, cyclization, and radial connection of the aggregates. After neutralizing the aqueous solution and removing the by-products before dialysis, all the samples are prepared from the neutralized aqueous solution unless otherwise noted. **a** TEM images showing the self-assembled evolution of POSS-SS-(PEG)_8_ with the concentration of 1 mg/mL in pH 8 solutions for various times. Insets are the correspondingly schematic structures. **b** SEM, **c** AFM, and **d** Cryo-TEM images of the monolayer vesicular formations at 12 h. Scale bars: 0.5 μm. **e** DLS results of the nanoparticles for various times. **f** The possible self-assembled mechanism of nanostructural bending, cyclization, and radial connection process. In this process, the longer cylinders can bend into the vesicles for conformational minimal energy. The whole formation process of the vesicles is clearly observed from the TEM images. On account of the limited space at the interior of the vesicles, the number, and density of disulfide bonds is significantly increased so that the possibility of thiol–disulfide exchange reaction is accordingly improved, gradually resulting in the radial connection to form the dense spheres. The orange cube represents the POSS core, the red ellipsoid represents the disulfide linkage and the cyan chain represents the PEG shell

Due to the flexible linkage and hydrophobic interactions among POSS molecules, these long and dense cylinders slowly bent into the loop to reduce the conformational free energy and finally generated the small vesicles (4–12 h, Fig. 5a). Careful observation found that the wall thickness of vesicles was approximately equal to the size of one POSS unit (1–2 nm). In other words, it feels like a lot of single POSS molecule standing side by side in a circle to form the wall of vesicle, presenting a monolayer vesicle, which were further testified by multi-techniques approaches (Fig. 5b–d). Supplementary Fig. 5 also provided the evidences to verify the nanoarchitectural bending and cyclization processes on the basis of hierarchical self-assembly behaviors. Besides, the gradual shrinkage of diameter in Fig. 5e indicated the increasing strutural compaction and the decreasing swelling property of these nanoaggregates. On account of the elevated density of disulfide bonds within the narrow mono-vesicles, thiol–disulfide exchange reaction had more possibilities to occur inside and induce the inner cross-connection toward the radial direction, and therefore the interior of vesicles became narrower and compacter until the formation of nanospheres at 16 h. The possibly self-assembled mechanism of nanostructural bending, cyclization, and radial connection process was depicted in Fig. 5f. MD simulations could illustrate the nanostructural bending and radial crosslinking pathways. From above simulation results, the cylinerical micelles presented some flexibility as the chain length is six units on account of the ductile methylene groups in the conjunction. So when the chain length reached tens or hundreds of repeat units, the cylinderical bending and cyclization would be inevitably produced and the aggregates transfered into the irregular patterns like shapeless or distorted vesicles. Because the S–S segment connected to POSS unit with all *trans* conformation is about 1 nm within the vesicles, the very short distance between two POSS centers led to the inner neighbor micelles crosslinked with each other, which further made the adjacent micelles closer in return. Therefore, the internal cavity of vesicles was slowly merged into the dense nanospheres, presenting a intramolecular crosslinking pathway.

### Periodically hierarchical self-assembly behaviors

**Fig. 6:**
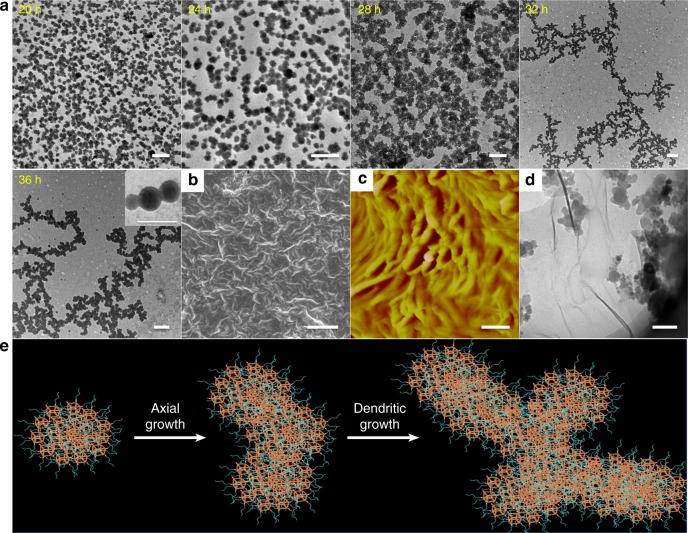
Hierarchically nanostructural orientation of axial and dendritic growth at microscales and macroscales. After neutralizing the aqueous solution and removing the by-products before dialysis, all the samples are prepared from the neutralized aqueous solution unless otherwise noted. **a** TEM images showing the self-assembled evolution of POSS-SS-(PEG)_8_ with the concentration of 1 mg/mL in pH 8 solutions for various times. The inset is the high magnification image of worm-like formations at 36 h, intuitively proved the interconnect arrangement of self-assemblies. **b** SEM, **c** AFM, and **d** Cryo-TEM images of the worm-like formations at 36 h. Scale bars: 0.5 μm. **e** The possible self-assembled mechanism of nanostructural growth with axial and dendritic directions. In this process, the large scale nanoparticles are still grown toward the axial orientation roughly at first, but finally formed the dendritic worm-like micelles with large size. The complex architectures, giant surface areas and reduced hydrophilic layers of the spheres provide more chances for the side-crosslinking to form the dendritic conformations. The orange cube represents the POSS core, the red ellipsoid represents the disulfide linkage and the cyan chain represents the PEG shell

Similarly to the unimolecular micelles, these nanospheres were still supposed to possess high superiority on the growth toward the axial orientation to form the longer and denser worm-like nanoparticles (Fig. 6a), which also ascribed to the high activity of the reactive points at the ends of these nanoparticles though they possessed the thick structures and large sizes. When the self-assembled evolution time reached 20 h, these nanospheres were interconnected with each other and transformed into the worm-like micelles. Further evolution, these worm-like micelles grew longer and thicker (Supplementary Fig. 6) and finally fromed the branch-like morphologies (24–32 h). The formation of dendritic aggregates may ascribe to the accumulation or crossover of the long worm-like micelles. Besides, it was inevitable to form the side-crosslinking micelles owing to their large surfaces and complex spatial stereostructures as well as the low amounts of the hydrophilic PEG chains. After 36 h, the formation of compact dendritic aggregates exhibited a kind of growth process by the dendritic pathway. SEM, AFM, and Cryo-TEM images (Fig. 6b–d) also presented the longer and denser morphologies with dendritic structures, further verifying the ongoing evolutive tendency of self-assemblies as well as the well-oriented growth pathway at microscopic scales (Fig. 6e).

**Fig. 7:**
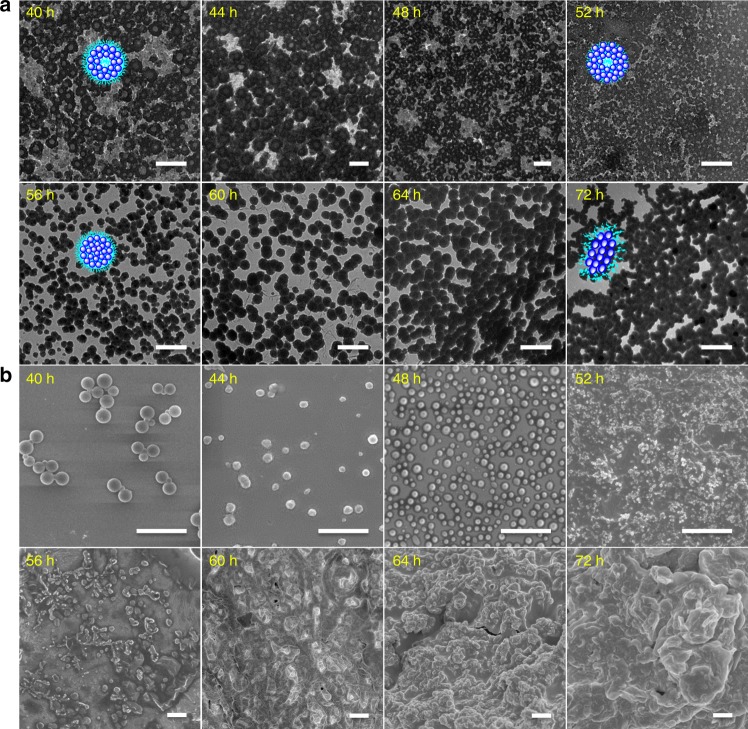
Hierarchically nanostructural bending, cyclization, and radial connection at microscales and macroscales. After neutralizing the aqueous solution and removing the by-products before dialysis, all the samples are prepared from the neutralized aqueous solution unless otherwise noted. **a** TEM and **b** SEM images showing self-assembled evolution of POSS-SS-(PEG)_8_ with the concentration of 1 mg/mL in pH 8 aqueous solutions for various times. Insets are the correspondingly schematic structures. Scale bars: 0.5 μm. The formation of hollow spheres verifies the nanostructural bending and cyclization mechanism. The reduced size of vesicle cavity demonstrates the inner circumferential crosslinking process, and the following spherical and elliptic aggregates confirm the periodically axial growth pathway even on the microscales and macroscales, which indicates the achievement of hierarchical self-assembly strategy for the guiding the morphology and size evolution from the nanoscopic solution aggregates to the microscopic and macroscopic hydrogel particles

Afterwards, the cyclization effects induced the evolution further into the hollow spheres (~50 nm of thickness, 40 h) for minimal energy, which also proceeded to the radial connection under the command of thermodynamics and dynamics (Fig. 7). The same as the previous stage of morphology and size evolution, the interior of hollow spheres gradually got compacter (44–52 h) as a result of effective exchange reaction within the extremely limited space, thus finally resulting in the formation of solid nanoparticles with the large sizes (56 h). Besides, the hollow spheres and solid nanoparticles were simultaneously found, convincingly verifying the ongoing evolutive tendency of self-assemblies and revealing the driving forces of shape-shifting by the hierarchical self-assembly behaviors. Repeatedly to former evolution, these large particles tent to accumulate together and further connected with each other to yield the ellipsoidal particles with preferentially axial growth directions (60–72 h). Without further morphology evolution, these ellipsoidal particles could maintain their structural stability within a period of time. It was mentioned that the floated fibrous straps were ultimately formed in water after 2 weeks, further verifying their preferentially axial growth behavors, which may reveal the actual hydrogel formation process from nanoscopic solution aggregates to macroscopic hydrogel particles.

**Fig. 8:**
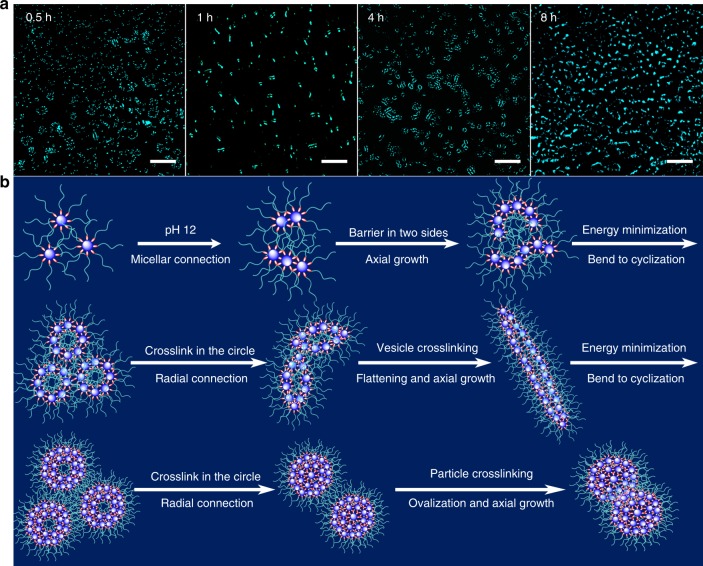
Hierarchical self-assembly strategy for morphology evolution within the full-scales. **a** Confocal laser scanning microscopy (CLSM) images of the POSS-SS-(PEG)_8_ @TPE-CHO aggregates in pH 12 THF/H_2_O mixtures for various times. Scale bars are 2 μm. On account of the cage structure of POSS units and propeller shape of TPE-CHO molecules, the POSS-SS-(PEG)_8_ @TPE-CHO aggregates could present a little difference of morphology evolution compared to the pure POSS-SS-(PEG)_8_ polymer after activation of thiol–disulfide exchange reaction. Nevertheless, the in situ morphology change from micelles to cylinders and then to vesicles was clearly monitored using the fluorescent marker with the prolongation of self-assembly time, intuitively demonstrating the hierarchical morphology evolution process. **b** Schematic illustration of the whole self-assembled evolution process of the POSS-SS-(PEG)_8_ polymer in aqueous solutions, including the possible formation mechanisms of these typical morphologies, such as unimolecular micelles, cylinders, vesicles, worm-like micelles, hollow spheres, dense and elliptic nanoparticles. The blue sphere represents the POSS core, the red ellipsoid represents the disulfide linkage and the cyan chain represents the PEG shell within the self-assemblies

For further verification of the hierarchically self-assembled evolution mechanism, in particular morphology changes from spherical micelles to cylindrical micelles and then to vesicles; we integrated the fluorescent markers into self-assembled skeletions to monitor the shape change. On account of the ever-improving hydrophobicity of the assemblies during the thiol–disulfide exchange reaction, aldehyde-functionalized TPE (TPE-CHO) was employed as a hydrophobic aggregation-induced emission (AIE) fluorogen to visually assess the morphology changes. Due to the tight packing of rigid POSS units and TPE segments in hydrophobic sections through strong π−π stacking, hydrogen bonding, and hydrophobic forces, the intramolecular rotation of TPE groups was restricted extremely in the limited space, thus emitting strong cyan fluorescence. Exactly as the predetermined shape transition mechanisms, these luminous self-assembled nanoparticles transferred from the spherical micelles to narrow cylinders to tortuous vesicles and then to complex micelle-linked cylinders (Fig. 8a), explicitly testifying this hierarchical self-assembly evolution towards the nanostructural axial growth, bending, and cyclization pathways with high priority.

From the above analysis, we can conclude that this self-assembled evolution was strongly relied on an efficient stepwise strategy for fabricating various nanostructures with a second level of hierarchy (Fig. 8b). By activation of thiol–disulfide exchange in alkaline solutions, the well-defined unimolecular micelles, based on the rigid backbone and special aggregation ability of POSS units, quickly aligned and oriented along the axial growth to form the cylindrical nanostructures. Further elongation and bending, the long cylinders transferred into monolayer vesicles for minimizing the free energy of systems. Afterwards, due to the reduced distance of reactive points and elevated concentrations of disulfides inside the vesicles, the internal crosslinking preferentially occurred like a radial connection to compact the vesicles. In views of this synchronous outer axial growth and inner circumferential crosslinking, these distorted vesicles were transformed into the thickly worm-like nanoparticles. After the following elongation, bending, and cyclization process of chain-like nanostructures, the dense hollow particles were generated by hierarchical self-assembly behaviors. Mentioned that the limited cavity furnished the unreactive points with more possibilities to accelerate the thiol–disulfide exchange reaction, and thus the small hollow particles quickly shrank inward and simultaneously elongated axially for ovalization to form the final ellipsoid particles. Therefore, by means of the pH-triggered thiol–disulfide exchange reaction on the surface or interior of nanostructures, an intriguing morphology and size evolution of self-assemblies was well-conducted under the guidance of hierarchical strategy for circulating axial growth, cyclization, and radial crosslinking process, which was universally applicable at all of nanoscopic, macroscopic, and microscopic scales.

### Investigation on exchange reaction and assembly conditions

Comprehensive analysis of this living thiol–disulfide exchange reaction in various conditions is beneficial for acquisition of unusual amphiphilic intermediates and creation of nanoarchitectures. To further study the exchange reaction and assembly condition-controlled morphology and size evolution, monomer concentration, reaction atmosphere, cosolvent effect, and UV switcher were taken into consideration for optimal regulation. Firstly, by reducing the polymeric concentration (0.1 mg/mL in pH 12) to slow down the reaction rate, the similar morphology evolution procedure including spherical micelles, cylinders, vesicles, worm-like micelles, hollow spheres, and elliptic nanoparticles was observed in Supplementary Fig. 7a, which indicated the strong POSS aggregation ability and the corresponding axial growth pathway on the microscales and macroscales were not relied on the concentration of POSS-containing polymers that was consistent with the previous report^57^. In addition to the arrangement of continuously shape-shifting nanostructures in aqueous solutions, we also utilized the mixed solvent of THF/H_2_O (*v*/*v* = 1:4, pH 12) to simultaneously retard the thiol–disulfide exchange reaction and weaken the POSS aggregation capacity. Thereinto, THF provided the POSS components with sufficient possibilities to gather together, so the cylinders could possess thicker backbones with slower axial growth for a period of time. The size and number increase of micelles could effectively reduce the interfacial area per chain and lower the free energy of system. Nevertheless, the steric hindrance effects of side PEG chains and the symmetrical cage structure of POSS units could still cause the oligomers to connect together with high priority at the axis direction and then undoubtedly bend into the loop to generate hollow spheres (Supplementary Fig. 7b). Without fast shape transition of inner circumferential crosslinking behavior, these hollow spheres were slowly interconnected with each other to form the larger aggregated clusters with concentrated, close-grained and hollow networks, which may be attributed to the severely reducing exchange reaction rate in THF environment although the distances of plentiful POSS centers were narrow within the hollow cavity. This result indirectly demonstrated the existence and significance of circumferential crosslinking process. Moreover, we changed the inert atmosphere (N_2_) and not found obvious difference of periodical transformation process, further proving the facile manipulation of thiol–disulfide exchange reaction and hierarchically nanostructural connection, axial growth, bending, and cyclization pathways (Supplementary Fig. 7c). Besides, on account of the dynamic disulfide bond that could also undergo exchange reaction with radical-mediated mechanism under UV irradiation, we adopted the photochemical method to adjust the reactive sites and regulate the UV-triggered self-assembly behaviors. In contrast to the thiolates-mediated exchange reaction with the driving force of selective core/shell separations, UV irradiation can damage one disulfide to form two freely thiyl radicals that induced the random radical-centered disulfide exchange reaction, giving rise to more intricately intermediate structures and multifarious morphologies. As shown in Supplementary Fig. 7d, the emergence and growth of cylinders were quickly formed under UV irradiation, followed by the generation of a very thick close-grained network as a result of UV-triggered multiple reactive sites and spatiotemporal precision for simultaneous multi-thiol–disulfide exchange reaction, but this random thiol–disulfide exchange mechanism led to the poor-selective core/shell separation and ill-defined morphlogy evolution. More importantly, UV irradiation could destroy the high-density disufides incidentally so that the thicker cylinder-linked networks were dissociated into a mass of shuttle-shaped sheets. Along with the concurrently random thiol–disulfide exchange and degradation reactions, prolonging irradiation time inconceivably resulted in the following square sheets, triangle-shaped plates, rhombus-shaped nanosheets, and silkworm particles, which revealed the great significances of selective core/shell separations on the tremendous contributions of reactive points at the ends with high-sufficient activity. These control experiments reflected that these pH-triggered disulfide reshuffling reaction and the corresponding self-assembly behaviors could be a facilely and efficiently hierarchical self-assembly strategy on guiding morphology and size evolution at nanoscales, microscales, and macroscales.

### Dissipative particle dynamics (DPD) simulation results

**Fig. 9:**
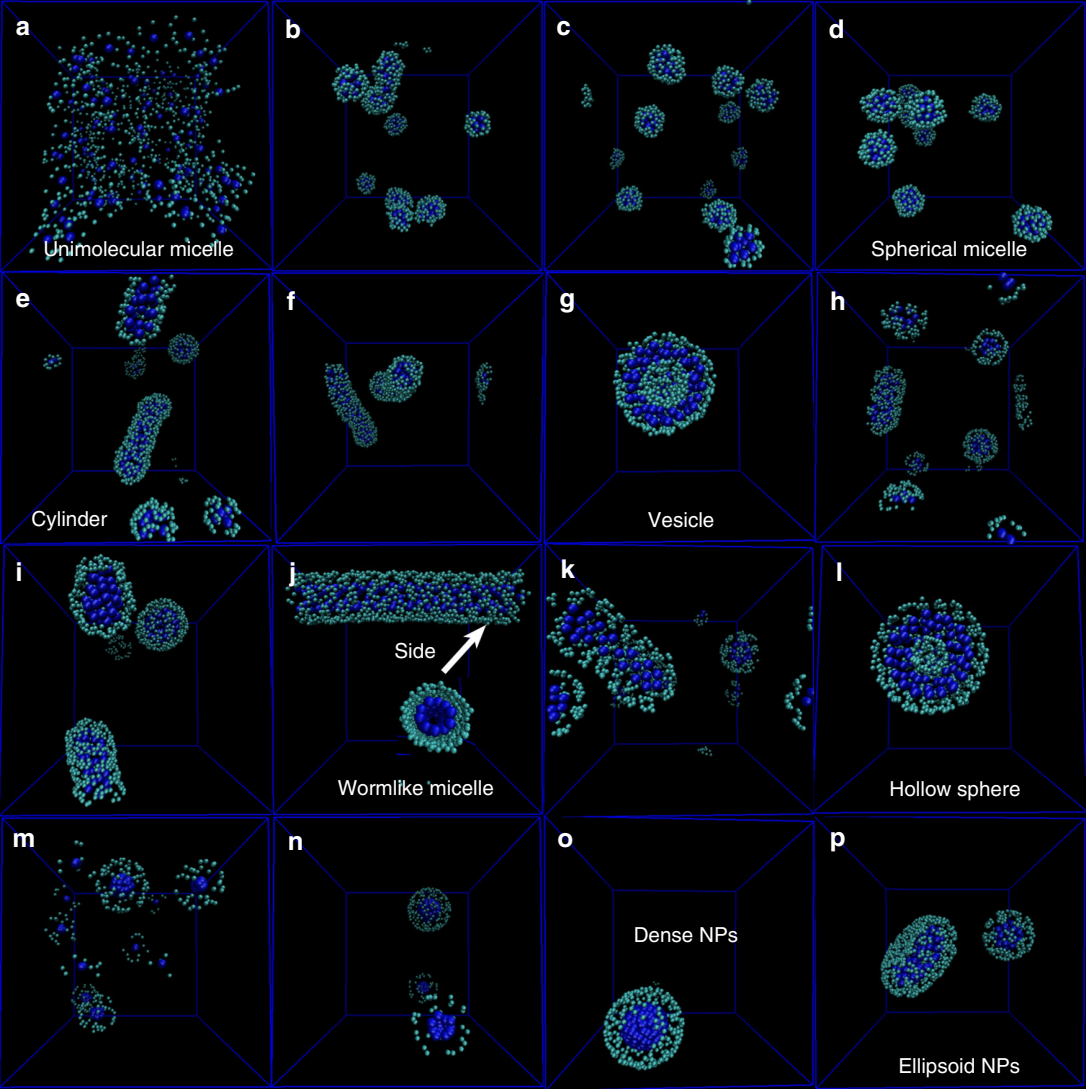
DPD simulations showing the formation pathway and morphology evolution of self-assemblies. **a** Unimolecular structure, **b**–**d** spherical structures, **e** cylindrical structures, **f** intermediate structures from cylinders to vesicles, **g** vesicular structures, **h**, **i** intermediate structures from vesicles to worm-like micelles, **j** long worm-like structures, **k** curved worm-like structures, **l** hollow spherical structures, **m**–**o** intermediate structures of dense nanoparticles, and **p** ellipsoid nanoparticle structures. The water beads are omitted for clarity (see Methods section for the detailed simulation parameters)

In fact, thiol–disulfide exchange reaction and self-assembly process occurred simultaneously, which was still a challenge to track in the experiments. Limited by the computational power, it was difficult to study the collective behavior of macromolecules with all-atom model. To overcome this challenge, we performed several coarse-grained simulations to better understand the self-assembly process. Here we employed DPD simulations to study the self-assembly behavior of POSS_*m*_-PEG_*n*_ oligomer, which is currently recognized as a viable simulation approach to intuitively study the macromolecular expression and formation pathway for morphology evolution^58–63^. Self-assembled structures can be quantitatively determined by the geometrical packing parameter *P*, where *P* = *v*/*a*_o_*l*_c_. *a*_o_ is the area of hydrophilic portions while *ν* and *l*_c_ are the volume and length of hydrophobic compartments, respectively. With the increase of *P*, the amphiphiles underwent the morphological transition from the unimolecular micelles to dense nanospheres. However, the conventional packing theory cannot interpret the formation of various morphologies in the self-assembly of POSS-(SS-PEG)_8_ polymer. As above mentioned, these assemblies were more abundant in contrast to those of traditional amphiphiles, which ascribed to that both the individual surfactant molecules and the assembled structures were dynamic during the self-assembly process. For a better understanding of self-assembly mechanism and intuitive observation of morphology transition, we performed DPD simulations using CG models in Supplementary Fig. 8. The morphology transferred from unimolecular to spherical micelles as the hydrophobic interaction increased between the POSS molecules (Fig. 9a–d). As the hydrophobic interaction improved, which was equal to the larger size of POSS-linked cores in the experiments, the cylindrical and vesicular structures appeared (Fig. 9e–g). This could be attributed to that the restriction of rigid POSS-embedded cores had a significant influence on the conformation of flexible chains, e.g., the stretching and compacting of PEG chains that yielded the diagram of varied assemblies. When the hydrophobic interaction between adjacent POSS-domains cores and water was further increased, the long worm-like micelles and hollow spheres occurred in Fig. 9h–l. Additionally, the topology constraint could also influence the shape transformation of aggregates. For example, the enough exposed surface area of POSS core was required if acquisition of other morphologies. As shown in Fig. 9m–o, dense nanospheres were obtained when the number of peripheral PEG chains reduced to a quarter. Further evolution, the elipsoid nanoparticles were finally formed in Fig. 9p. These DPD simulations further testified the process of morphology evolution in theory.

### pH-Switched morphology evolution with an on/off function

**Fig. 10:**
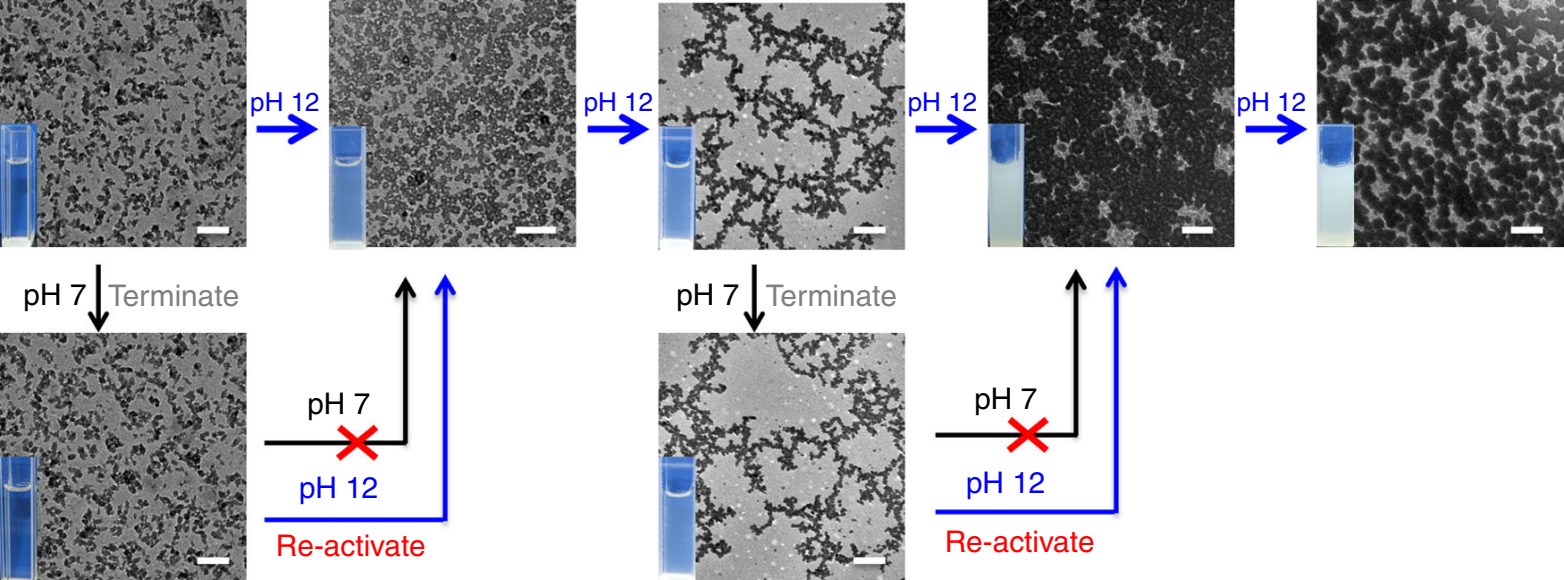
pH-Switched morphology evolution. TEM images showing in situ morphology evolution with termination and reactivation function under the control of system pH. Scale bars: 0.5 μm. During the whole morphology transition process, once the thiol–disulfide exchange reaction is interrupted by neutralization at any time, the self-assemblies, including cylinders, vesicles, worm-like micelles, hollow spheres, etc. at that moment are fixed and kept stable for more than 7 days, but this morphology and size evolution is easily restarted and returned back on its right track by reactivation of the exchange reaction at pH 12 solutions. This whole self-assembled evolution process can be easily activated, controllably terminated and interrupted, and reinitiated by the external pH stimuli whenever needed. The inset shows that the assembled solution in the quartz plate is changed from transparency to turbidity

Once the thiol–disulfide exchange was interrupted at any moment by neutralizing the system pH, the thiolates of assemblies were deactivated to thiols, thus no further exchange reaction was conducted any more as well as the termination of hierarchical self-assembly behaviors. However, these stable inert thiols and trapped morphology evolution could be reactivated whenever needed. For instance, when the polymer solution was basified at pH 12 (Fig. 10), the short cylinders were formed around 30 min later and further transferred into vesicles after 2 h. By neutralizing solutions at pH 7, this transition could be interrupted and the cylindrical micelles were kept stable for a long time. However, after rebasification of system pH to 12, the morphology transition was restarted to evolve into the vesicles. Similarly, the stable worm-like micelles could also be yielded by interruption of morphology evolution in aqueous solutions. These neutralized self-assemblies had a good long-term stability and basically maintained their shapes and sizes at least 7 days in mild aqueous environments. These results indicated a simple method to differentiate and gain the complex and intermediate morphologies. Therefore, the conceivable self-assemblies with customized structures and properties can be easily tailored only by the control of system pH within a predetermined time.

### Importance of uniform core on the hierarchical behaviors

According to the aforementioned analysis, we conjecture that this living hierarchically assembled strategy for self-assembled evolution should be recognized as a versatile method because it may also be suitable for other materials with similar disulfide-linked dendritic architectures. To validate this proposal, we produced a disulfide-linked hyperbranched poly(BAC2-AEPZ1)-PEG polymer with an irregular core by Michael addition polymerization (Supplementary Fig. 9)^46^. As seen in Supplementary Fig. 10, a scattered anisotropic variation of assemblies, mainly driven by the hydrophobic interactions, were conducted in alkaline solutions. The simple axial growth was observed in the first stage on account of steric hindrance areas from hydrophobic domains and the swollen layers from the stretched PEG shells at the middle of micelles. However, as a result of random linkages by the newly-formed disulfides among the nanoparticles, hierarchical self-assembly failed to perform the cylindrical bending and circumferential crosslinking behaviors, which indicated the importance of the uniformity and rigidity of dendritic core. To further verify this principle, we conducted another control experiment using a well-defined but asymmetric cyclodextrin (*β*-CD) core to obtain the *β*-CD-(SS-PEG)_3-4_ polymer via an acylation reaction (Supplementary Fig. 11), which self-assembled into unimolecular micelles at the original stage and further transferred into the worm-like micelles in pH 12 solutions. On account of the uniform size and highly crosslinking CD cores, these aggregates performed the unequivocal step-change shifts in cylindrical micelle length and population during the process of thiol–disulfide exchange reaction. The stacking *β*-CD molecules played an important role in the micellar dimensional growth. As shown in Supplementary Fig. 12, the sizes increased from unimolecule scale to nanoscale to micrometer scale in alkaline solutions. However, the cyclization and radial crosslinking process were not observed due to the asymmetrical cone, ring tensions, and huge steric hindrance of the cyclodextrin, which further suggested the significance of symmetry on the sequential morphlogy transition. Nevertheless, the living hierarchical self-assembly strategy for size evolution was indeed effective and universal, because once neutralizing the solution, the size growth was interrupted and fixed with a long-term stability. Therefore, different types of polymeric assembly evolution with an on/off function can be easily customized via design of disulfide-linked architectures with uniform and nondeformable cores by means of the living thiol–disulfide exchange reaction.

### Potential application for drug delivery systems

High loading efficiency and stimuli-responsive property are two pivotal factors for creation of robust nanocarriers. In views of the increased hydrophobicity/hydrophilicity ratio, varied molecular topology and elevated number of disulfide linkages during the morphology transition process, this living self-assembly system was of great significance on the fabrication of redox-responsive drug delivery systems. The encapsulation ability was firstly quantitatively estimated using fluorescent TPE probes in pH 12 solutions. When the AIE-active TPE-CHO molecule was added into the THF/H_2_O (*v*/*v* = 1:4) mixtures of POSS-(SS-PEG)_8_ polymer, the activation and process of morphology evolution led to the gradual encapsulation of more and more hydrophobic TPE molecules into the assemblies along with the growth of fluorescent intensity (Supplementary Fig. 13). After incubation for 24 h, the hydrophobicity of aggregates was intensely enhanced so that the precipitates occurred and gradually increased at the bottom and the luminescence of upper clear solution was substantially weakened corresponding to the sharp decline of fluorescent intensity. After sufficient incubation time for 6 days, the separated white precipitates emitted strong cyan fluorescent light while the supernatant liquid was nearly non-emissive, suggesting the complete encapsulation of TPE-CHO into polymeric assemblies.

As for the encapsulation of antitumor drugs, taking account of the incompatible alkaline environment for protein and live cells, we changed into pH 7.4 to in situ trigger the thiol–disulfide exchange reaction though it owned a very low reaction rate. Nevertheless, the drug-loading efficiency could be still up to 70% (Supplementary Fig. 14a) along with the increase of hydrophobic/hydrophilic ratio and variation of molecular topology. Similarly to the pH-switched self-assembly behaviors, the drug-loaded efficiency also possessed a great correlation with thiol–disulfide exchange reaction in various pH conditions, which could be facilely activated, controllably terminated and interrupted, and reinitiated by the external stimuli (Supplementary Fig. 14b).Then we filtered out two representative DOX-loaded micelles to estimate the intracellular drug delivery after comprehensive considerations of particle size, DTT-triggered release behavior, cell viability, and antitumor activity toward MCF-7 cells (Supplementary Figs. 14c, d and 15a, b). Confocal laser scanning microscopy exhibited the cellular uptake and intracellular drug release behaviors of DOX-loaded aggregates (Supplementary Fig. 15c). The enhanced fluorescent intensity revealed the rapid internalization of drug carriers and efficient release of antitumor DOX drugs into the nucleus. Accordingly, the above unique advantages including high drug-loading efficiency, smart redox-responsibility, and good biocompatibility indicated the great potential biological applications in drug delivery system.

## Discussion

In summary, we demonstrated a tunable manipulation of solution assemblies evolution in situ based on the living hierarchical self-assembly of amphiphilic POSS-(SS-PEG)_8_ polymer. Careful tailor of the pH-triggered thiol–disulfide exchange reaction and self-assembled conditions could make the reactive points at the ends of these nanoparticles with high activity, thus presenting the intriguing morphology and size evolutions like periodically micellar connection, axial growth, bending and cyclization processes from the initially unimolecular pre-assemblies on the stepwisely self-assembly behaviors, by which the multifarious morphologies like spherical micelles, cylinders, vesicles, worm-like micelles, hollow spheres, and elliptic nanoparticles as well as the gradual size growths were formed in sequence. Such controllable strategy to guide the hierarchical self-assembled evolution was applicable from the nanoscopic assemblies to microscopic and macroscopic aggregates. Notably, this hierarchical self-assembled evolution could be interrupted and reactivated at any moment only by removal of the external stimuli, and thus the desirable morphology and size was facilely gained with good stability, which provided unique advantages and more opportunities to fabricate the intelligent drug carriers with high loading efficiency and stimuli-responsive property. This hierarchical concept to program the dynamic morphology and size evolution is also universal for other disulfides-linked dendritic polymers with well-structured cores, which will provide useful guidance for achieving more well-worth exploring self-assembly mechanism and artificial superstructures with defined shapes and desired functions.

## Methods

### Materials and synthesis

(3-Mercaptopropyl) trimethoxysilane (97%, J&K), 2,2'-dithiodipyridine (98%, Energy Chemical), poly(ethylene glycol) methyl ether (PEG-OH, *M*_n_ = 750 g/mol, 98%, Energy Chemical), thionyl chloride (97%, J&K), 2-mercaptoethylamine hydrochloride (MCA, 99%, Energy Chemical), 1-(2-aminoethyl)piperazine (AEPZ, 97%, Aldrich), diisopropanolamine (DIAD, 98%, Energy Chemical), phthalimide (97%, Energy Chemical), triphenylphosphine (99%, Energy Chemical), hydrazine hydrate (95%, J&K), triethylamine (TEA, 99%, Beijing Chemical Works), succinic anhydride (99%, J&K), *N, N'*-bis(acryloyl)cystamine (BAC, 98%, Fluka), *β*-CD ( > 95%, TCI) was dried at 70 °C under vacuum for 24 h before use. Cell counting kit-8 was purchased from Beyotime Biotechnology. DMEM (Corning) with 4.5 g /L glucose, l-glutamine, without sodium pyruvate was purchased from Beijing BioDee Biotechnology Co. Ltd. MCF-7 cell lines were purchased from the American type Culture Collection (ATCC, Rockville, MD). Other reagents were commercially obtained from Beijing Chem. Co. and used without further purification.

### Synthesis of the PEG-NH_2_ polymer

A solution of DIAD (50 mmol, 10.7 g) in 20 mL of DCM was added dropwise to a solution of PEG (*M*_n_ = 750 g /mol, 25 mmol, 18.75 g), phthalimide (50 mmol, 7.43 g), and triphenylphosphine (50 mmol, 13.25 g) in 70 mL of DCM at room temperature. The solution was stirred at room temperature for 7 days. The solution was removed under reduced pressure. The product was then dissolved in water and removed by filtration and the aqueous solution was washed with diethyl ether. Water was removed under reduced pressure and the oily product of PEG-phthalimido was dried at 40 °C under high vacuum with 99% yield. ^1^H NMR (CDCl_3_, 400 MHz, ppm): δ 3.38 (s, 3 H), 3.73 (t, 2 H), 3.83 (t, 2 H), 7.84 (m, 4 H). ^13^C NMR (CDCl_3_, 100 MHz, ppm): δ 40.6, 59.2, 65.3, 70.4, 71.6, 123.7, 132.2, 168.1.

PEG-phthalimido (25 mmol) in 100 mL of ethanol were treated with 500 mmol of aqueous solutions of hydrazine hydrate under reflux overnight. Concentrated hydrochloric acid was added to the cold solution up to pH 2 and the precipitate was removed by filtration. After evaporation of ethanol, a small amount of water was added to the extracts and aqueous sodium hydroxide was added up to pH 10. The polymers were extracted with DCM for several times. The solution was dried over MgSO_4_ and after evaporation to obtain the oily PEG-NH_2_. Water dilution followed by washing with diethyl ether, and evaporation of water were necessary to fully purify the PEG-NH_2_ at 40 °C under high vacuum with 95% yield. ^1^H NMR (CDCl_3_, 400 MHz, ppm): δ 3.11 (t, 2 H), 3.38 (s, 3 H), 3.67 (t, 2 H). ^13^C NMR (CDCl_3_, 100 MHz, ppm): δ 41.6, 59.3, 70.1, 70.4, 71.6, 73.1.

### Synthesis of the PEG-SH polymer

PEG-OH (*M*_n_ = 750 g /mol, 7.5 g, 10 mmol) and triethylamine (7 mL, 50 mmol) were dissolved in 100 mL of anhydrous DCM and cooled down to 0 °C for 15 min. Methanesulfonyl chloride (4 mL, 50 mmol) was then added to the mixture. After stirring for 5 h at room temperature, the mixture was quenched with 150 mL of water, and extracted with 50 mL of DCM for several times. Then the combined organic layers were washed with water and dried over MgSO_4_ to obtain the yellow product of PEG-SO_3_CH_3_ with 99% yield. ^1^H NMR (CDCl_3_, 400 MHz, ppm): δ 3.15 (s, 3 H), 3.38 (s, 3 H), 3.71 (t, 2 H). ^13^C NMR (CDCl_3_, 100 MHz, ppm): δ 38.2, 59.2, 67.8, 69.2, 70.4, 72.1.

As-synthesized PEG-SO_3_CH_3_ (8.2 g, 10 mmol) and potassium thioacetate (3.6 g, 25 mmol) were dissolved in 100 mL of anhydrous THF under a nitrogen atmosphere. The reaction mixture was degassed three times with N_2_ and then heated to 60 °C. After stirring for 12 h, the mixture was quenched by water and extracted with DCM. The DCM layer was collected, and residual water was removed with MgSO_4_ to obtain the yellow product of PEG-SCOCH_3_ with 89% yield. ^1^H NMR (CDCl_3_, 400 MHz, ppm): δ 2.31 (s, 3 H), δ 3.01 (t, 2 H), 3.38 (s, 3 H), 3.71 (t, 2 H), 4.06 (t, 2 H). ^13^C NMR (CDCl_3_, 100 MHz, ppm): δ 30.5, 34.5, 59.3, 70.4, 71.8,195.0.

As-synthesized PEG-SCOCH_3_ (6.9 g, 8.4 mmol) was dissolved in methanol under a N_2_ atmosphere and degassed three times. Concentrated hydrochloric acid (0.8 mL) was added to the solution up to pH 2. The mixture was refluxed at 80 °C for 24 h, quenched with water, and then extracted with DCM. The DCM layer was collected, and residual water was removed by MgSO_4_ to obtain the final white product of PEG-SH with 82% yield. ^1^H NMR (CDCl_3_, 400 MHz, ppm): δ 1.56 (s, 3 H), δ 2.69 (t, 2 H), 3.38 (s, 3 H), 3.81 (t, 2 H). ^13^C NMR (CDCl_3_, 100 MHz, ppm): δ 24.1, 59.1, 70.1, 70.4, 71.8, 72.9.

### Synthesis of the star-shaped POSS-(SS-PEG)_8_ polymer

Octa (3-mercaptopropylsilsesquioxane), POSS-(SH)_8_, was synthesized as follows: 50 mL of methanol, 10 mL of acetonitrile, and 8 mL of concentrated hydrochloric acid were added into a 100 mL flask and mixed to get a heterogeneous solution under the atmosphere of nitrogen. Then, (3-Mercaptopropyl) trimethoxysilane (8.5 g, 40 mmol) was added into the solution. The mixture was refluxed at 80 °C for 5 days to produce white precipitates. The crude products were obtained after filtration, washing with cold methanol, and drying. Then recrystallization from acetone afforded the product as white solids with 18% yield. ^1^H NMR (CDCl_3_, 400 MHz, ppm): δ 0.75 (t, 2 H), 1.35 (s, 1 H), 1.75 (m, 2 H), 2.53 (q, 2 H). ^13^C NMR (CDCl_3_, 100 MHz, ppm): δ 10.8, 27.1, 27.4. ^29^Si NMR (CDCl_3_, 300 MHz, ppm): δ 34.9.

PEG-SS-2TP polymer was synthesized as follows: PEG-SH (10.2 g, 13.3 mmol) was dissolved in 50 mL of methanol and added dropwise to a stirred solution of 2, 2′-dithiodipyridine (8.8 g, 50 mmol) dissolved in 40 mL of methanol. The reaction was kept under an argon atmosphere to minimize free thiol oxidation. After 3 days, the mixture was concentrated under reduced pressure. The product was precipitated by addition of 50 mL of cold ether and purified by redissolving in 10 mL of methanol and precipitating into 50 mL of cold ether three times to give a white powder. The final product of PEG-SS-2TP was obtained in vacuo with 80% yield. ^1^H NMR (CDCl_3_, 400 MHz, ppm): δ 2.99 (t, 2 H), 3.37 (s, 3 H), 3.82 (t, 2 H), 7.08-8.45 (m, 4 H). ^13^C NMR (CDCl_3_, 100 MHz, ppm): δ 38.3, 58.5, 69.6, 70.5, 71.6, 71.8, 120.6, 121.9, 129.1, 149.2 159.6.

POSS-(SS-PEG)_8_ polymer was synthesized as follows: POSS-(SH)_8_ (0.89 g, 0.88 mmol) was dissolved in 50 mL of DCM and added dropwise to a stirred solution of PEG-SS-2TP (9.31 g, 10.6 mmol) dissolved in 50 mL of DCM. The reaction was kept under nitrogen atmosphere to minimize free thiol oxidation. The reaction mixture was stirred at 40 °C for 6 days and then concentrated by rotary evaporation to yield a viscous liquid. The product of POSS-(SS-PEG)_8_ was purified by ultrafiltration (MWCO 2000) and collected after freeze-drying with 68% yield. ^1^H NMR (CDCl_3_, 400 MHz, ppm): δ 0.73 (t, 2 H), 1.76 (m, 2 H), 2.41 (t, 2 H), 2.83 (t, 2 H), 3.37 (s, 3 H). ^13^C NMR (CDCl_3_, 100 MHz, ppm): δ 10.7, 22.0, 37.8, 41.3, 58.8, 68.6, 69.6, 69.8, 70.5, 71.8.

### Synthesis of hyperbranched poly(BAC2-AEPZ1)-PEG polymer

AEPZ (0.4 g, 3 mmol) was dissolved in 5 mL of methanol and dropwise added into 20 mL of methanol containing the BAC (1.58 g, 6.0 mmol). The solution was stirring at 50 °C for 6 days, and then PEG-NH_2_ (3.46 g, 4.6 mmol) was added and further kept stirring at 60 °C for another 5 days to seal terminal vinyl groups. The reaction mixture was concentrated by rotary evaporation and precipitated in 100 mL of cold ether for three times. Thereafter, the target polymer was collected and purified by redissolving in 10 mL of methanol and precipitating into 200 mL of acetone containing 10 mL of 37% concentration HCl. The final product of poly(BAC2-AEPZ1)-PEG polymer (*M*_n_ = 1.9 kDa, PDI = 2.0) was obtained in vacuo at 50 °C overnight.

### Synthesis of multi-arm CD-(SS-PEG)_3-4_ polymer

*β*-CD (1.14 g, 1 mmol) and Et_3_N (0.7 mL, 5 mmol) was dissolved in 25 mL of dry DMF and added dropwise to a stirred solution of PEG-SS-COCl (3.2 g, 4 mmol) dissolved in 20 mL of DMF. The reaction mixture was stirred at 40 °C for 6 h and then concentrated by rotary evaporation to yield a viscous liquid. The product, CD-(SS-PEG)_*n*_ (*n* = 3–4, 3.15 g, 80%), was purified by ultrafiltration (MWCO 2000) and collected after freeze-drying.

### pH-triggered morphology and size evolution in water

A total of 5 mg of POSS-(SS-PEG)_8_ was dissolved in 5 mL of deionized water with 10 μL of 5 M NaOH was added to adjust pH to 12 or 8. Then 0.5 mg of cysteamine was mixed to trigger the reaction. After standing for a certain time, the stable morphology can be obtained by neutralization with 0.2 mL of saturated NH_4_Cl solution, and the morphology evolution was obtained from the solution samples at a predetermined time. Similar to the above preparation process for POSS-(SS-PEG)_8_ polymer with the concentration of 0.1 mg/mL in pH 12 solutions, its morphology evolution was also obtained from the solution samples at a predetermined time.

The self-assembly process of hyperbranched poly(BAC2-AEPZ1)-PEG and CD-(SS-PEG)_3–4_ polymers were as same as that of POSS-(SS-PEG)_8_ polymer with the concentration of 1 mg/mL in pH 12 solutions, After standing for a certain time, the stable morphology can be obtained by neutralization with 0.2 mL of saturated NH_4_Cl solution, and the morphology evolution was obtained from the solution samples at a predetermined time.

### pH-triggered morphology and size evolution in THF/water

A total of 5 mg of POSS-(SS-PEG)_8_ was dissolved in 5 mL of THF/water (*v*/*v* = 1:4) with 10 μL of 5 M NaOH was added to adjust pH to 12. Then 0.5 mg of cysteamine was mixed to trigger the reaction. After standing for a certain time, the stable morphology can be obtained by neutralization with 0.2 mL of saturated NH_4_Cl solution and morphology evolution was obtained from the solution samples at a predetermined time.

### UV-triggered morphology and size evolution in water

A total of 5 mg of POSS-(SS-PEG)_8_ was dissolved in 5 mL of deionized water with 0.5 mg of cysteamine and 0.1 mg of IR2959. Then irradiation triggered the reaction under a UV lamp (300 W, 200–400 nm) with gentle rotation by using a mechanical agitator to ensure homogeneity. After standing for a certain time, the stable morphology can be obtained by removal of the UV irradiation, and the morphology evolution was obtained from the solution samples at a predetermined irradiation time.

### Preparation of POSS-(SS-PEG)_8_@TPE aggregates

Various POSS-(SS-PEG)_8_@TPE aggregates were prepared as follows: 5 mg of POSS-(SS-PEG)_8_ and 1 mg of TPE-CHO molecules were firstly dissolved in 1 mL of THF, then 4 mL of ultrapure water was added dropwise into the solution at the rate of 0.05 mL/min via a syringe pump. Then 10 μL of 5 M NaOH was added to adjust pH to 12. Then 0.5 mg of cysteamine was mixed to trigger the reaction. After standing for a certain time, the stable morphology can be obtained by neutralization with 0.2 mL of saturated NH_4_Cl solution, and the morphology evolution was obtained from the solution samples at a predetermined time. Then the photograph, CLSM image and fluorescent intensity of various TPE-loaded aggregates were measured by fluorescence measurements.

### Preparation of POSS-(SS-PEG)_8_@DOX aggregates

Various POSS-(SS-PEG)_8_@DOX aggregates were prepared as follows: 5 mg of POSS-(SS-PEG)_8_ was dissolved in 1 mL of DMF, followed by adding a predetermined amount of DOX∙HCl and 1.5 molar equiv of triethylamine and stirred at room temperature for 2 h. Then 4 mL of PBS (pH 7.4, 8, or 12) was added dropwise into the solution at the rate of 0.05 mL/min via a syringe pump. After triggering the exchange reaction for a certain time, the stable morphology can be obtained by neutralization with saturated NH_4_Cl solution and morphology evolution was obtained from the solution samples at a predetermined time. Subsequently, the solution was dialyzed against deionized water for 24 h (MW cutoff, 4 kDa) to remove free DOX and by-products, followed by lyophilization to obtain the freeze various dried DOX-loaded aggregates.

### In vitro DOX release

A series of DOX-loaded POSS-(SS-PEG)8 aggregates with various thiol–disulfide exchange reaction times were added into a dialysis membrane tube (MW cutoff, 4 kDa) and then immersed into 30 mL of pH 7.4 PBS containing 10 mM DTT in a shaking water bath at 37 °C. The POSS-(SS-PEG)_8_@DOX solutions were lyophilized and then dissolved in DMF to determine the total loading of DOX by the fluorescence spectroscopy. The amount of DOX release was determined using UV-vis absorbance (excitation at 480 nm). All the DOX release experiments were conducted in triplicate and the results were expressed as the average ± standard deviation.

### Cellular uptake and intracellular localization

MCF-7 cells were seeded onto a 96-well plate at a density of 5 × 10^4^ cells/well in Dulbecco’s Modified Eagle Medium (DMEM) containing 10% fetal bovine serum (FBS). After incubation for 24 h (37 °C, 5% CO_2_), the medium was removed and replenished by the fresh DMEM containing 10% FBS. Then the prescribed amounts of free DOX, POSS-(SS-PEG)_8_@DOX-8 h, and POSS-(SS-PEG)_8_@DOX-36 h aggregates were added. After the incubation of cells for various times, the culture medium was removed and the cells were washed with PBS for several times. Afterwards, the cells were fixed with 4% paraformaldehyde at room temperature and the slides were rinsed with PBS for three times. Finally, the MCF-7 cells were directly observed using a confocal laser scanning microscopy (CLSM) with the excitation of 488 nm.

### Activity analyses

The cytotoxicity of POSS-(SS-PEG)_8_ and POSS-(SS-PEG)_8_@DOX aggregates (exchange reaction at pH 7.4 for 8 and 36 h) was evaluated in vitro by CCK-8 assay. MCF-7 cells were seeded in 180 μL of DMEM containing 10% FBS at a density of 1 × 10^4^ cells/well. After incubation for 24 h (37 °C, 5% CO_2_), the medium was replaced by 90 μL of fresh DMEM containing 10% FBS, and then 20 μL of samples with various concentrations of nanoparticle suspensions in PBS (pH 7.4) were added and further incubated for another 24 h. After removal of the culture medium, 100 μL of fresh DMEM and 10 μL of CCK-8 kit solutions were immediately added and homogeneously mixed. After incubating the cells for 4 h in a CO_2_ incubator, 100 μL of reaction solutions were put into a 96-well plate. The optical density of each well at 450 nm was carefully measured by a microplate reader. Cells cultured in DMEM containing 10% FBS without pretreatment were used as the control. The resulting data were expressed as the average ± standard deviation (*n* = 5).

### All-atom MD simulations

All the simulations were carried out using GROMACS. The force field of POSS_*m*_-PEG_*n*_ oligomer were from refs. ^62^^,^^63^. The TIP3P water model was used for water. In MD simulations, one POSS_*m*_-PEG_*n*_ oligomer with different degree of polymerization was solvated in water box. The details of box size are listed in Supplementary Table 1. These systems were firstly simulated at 300 K and 1 atm for 20 ns. Then 1 μs production simulations were performed. The time step is 2 fs. The temperature and pressure were maintained by berendsen thermostat and barostat in equilibrium and in production simulation the Nose–Hoover thermostat and Parrinello-Rahman barostat were used. Electrostatic interactions were calculated using particle mesh Ewald and a real space cutoff of 1.2 nm. van der Waals interactions were switched to zero between 1 nm and 1.2 nm. Three independent MD runs were conducted for every system.

### DPD simulations

To understand the self-assembly behaviors of POSS-(SS-PEG)_8_ polymer in water, some DPD simulations were performed by constructing a specific coarse-grained (CG) model of POSS-(SS-PEG)_8_ polymer wherein the eight branches were attached to one big CG POSS bead. As shown in Supplementary Fig. 8, there were two types of beads, i.e., P and G for POSS and PEG. The atomistic model was coarse-grained according to the molecular segment size in all-atom simulation. In this study we adjusted the size of central POSS bead to simulate the polymerization of POSS-domain cores. The interaction parameter a_ij_ between POSS and PEG beads was also increased to describe the dynamic hydrophobic interaction.

Within the CG model, neighboring CG beads are bonded to each other by a harmonic spring potential, *U*_b_ = 0.5 *k*_b_(*r*−*r*_0_)^2^, where *k*_b_ is the spring constant and *r*_0_ is the equilibrium bond length. In this study, *k*_b_ = 25 and *r*_0_ = 0.7. The concentration of POSS-(SS-PEG)_8_ polymer in the solution $\varphi$ is 0.05, which is calculated by the following formula,

1 $$\varphi = \frac{{N_{\rm{P}}V_{\rm{P}} + N_{\rm{G}}V_{\rm{G}}}}{{N_{\rm{P}}V_{\rm{P}} + N_{\rm{G}}V_{\rm{G}} + N_{\rm{W}}V_{\rm{W}}}}$$

where *N*_P_, *N*_G_, and *N*_W_ are the number of POSS, PEG, and water beads while *V*_P_, *V*_G_, and *V*_W_ are the volume of one bead of POSS, PEG, and solvent, respectively.

The simulations were performed in NVT ensemble with periodical boundary conditions and at a fixed system number density of 3.0. All the CG beads have the same mass as *m* = 1. The interaction cutoff radius is set to 1 as the unit of length. The reduced temperature is 1.0. The size of simulation box is 30×30×30. In the DPD simulations the modified velocity-verlet algorithm was used to integrate the equations of motion with a time step of 0.03. The simulation systems were pre-equilibrated with 1 × 10^6^ steps by setting *a*_*ij*_ = 25 and *T* = 1.0 for all DPD beads. Then we performed another simulation with 2 × 10^6^ steps to observe the morphology evolution of aggregates.

General characterization. ^1^H, ^13^C, and ^29^Si NMR spectra were recorded on a Bruker DRX-400 spectrometer using deuterated chloroform (CDCl3) as the solvent and tetramethylsilane (TMS) as the internal standard. Transmission electron microscopy (TEM) studies were performed with a JEM-2200FS instrument (JEOL, Japan). Cryogenic transmission electron microscopy (Cryo-TEM) studies were examined in a cryogenic sample holder (Gatan 626) with a JEM-2200FS at approximately −174 °C. Scanning electron microscopy (SEM) images were recorded on a JSM-6700F microscope (JEOL, Japan) with the acceleration voltage of 5 kV. Atomic force microscope (AFM) images were obtained with a Nanoscope Multimode III AFM instrument (Vecco, America) in tapping mode. Dynamic light scattering (DLS) profiles were measured on a commercial laser light scattering spectrometer (ALV/DLS/SLS-5022F) equipped with a multi-τ digital time correlator (ALV5000) and a cylindrical 22 mW UNIPHASE He–Ne laser (*λ*_0_ = 632.8 nm). Confocal laser scanning microscopy (CLSM, Zeiss LSM 510, Germany) images were obtained under the excitation at 488 nm. UV-vis spectra were recorded by a Hitachi U-3010 spectrometer and fluorescence measurements were performed on a Hitachi F4600 photoluminescence spectrometer with a xenon lamp as a light source.

### Data availability

The authors declare that the data supporting the conclusions of this study are available within the article and its Supplementary Information file or from the corresponding author upon reasonable request.

## Electronic supplementary material

Supplementary Information
