# Supplementary material for: Dynamic and programmable morphology and size evolution via a living hierarchical self-assembly strategy
Source: Nat Commun. 2018 Jul 17;9:2772. doi: 10.1038/s41467-018-05142-3 (PMC6050331; doi:10.1038/s41467-018-05142-3)
Supplement: Supplementary file 1 — Supplementary Information [file 41467_2018_5142_MOESM1_ESM.pdf]

Dynamic and programmable morphology and size evolution via a living hierarchical self-assembly strategy

Wang et al.

Supplementary Figures and Tables

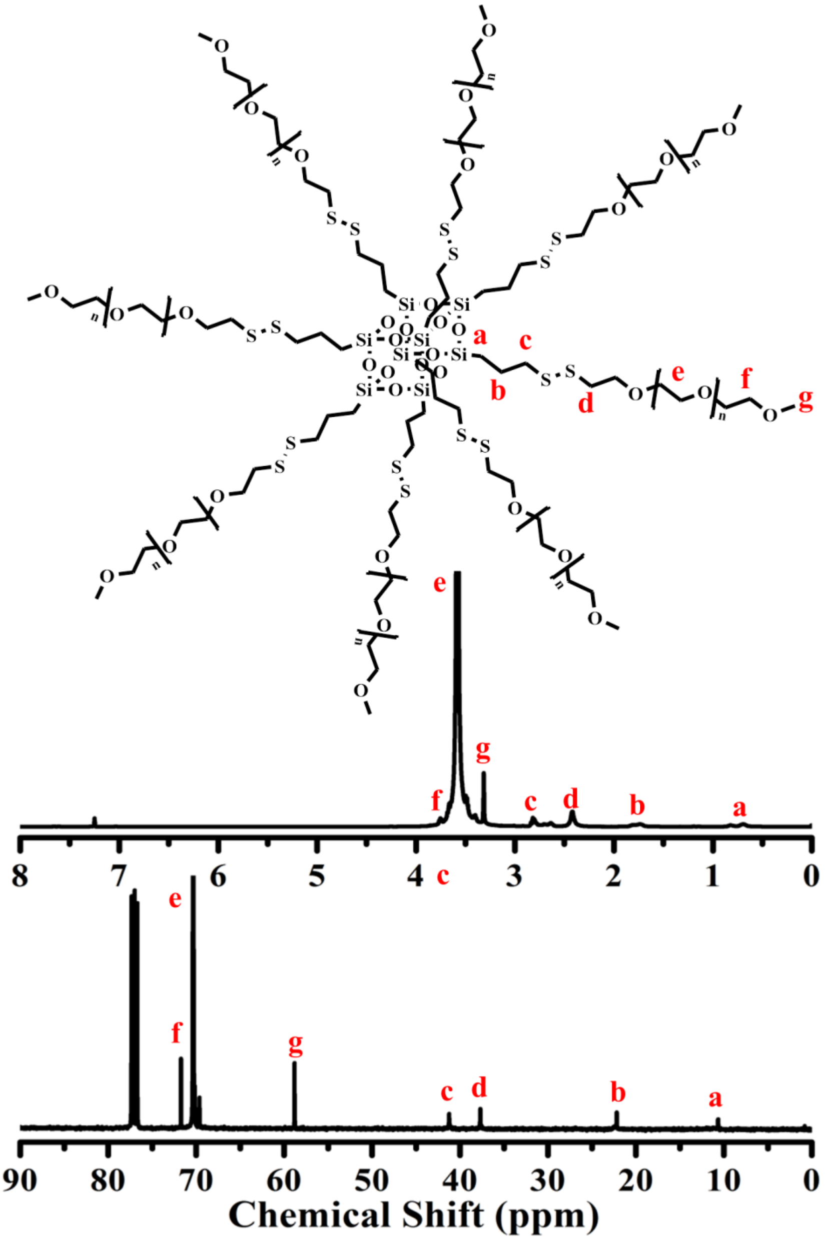

Supplementary Figure 1. ^{1}H and ^{13}C NMR spectra of POSS-(SS-PEG)$_8$ at $CDCl_3$. The signals at δ 0.73, 1.76 and 2.41 ppm belonged to the protons of $-CH_2$ groups adjacently to POSS unit while the resonance peaks at δ 3.3 and 3.5-4.1 ppm were assigned to the PEG chain in the ^{1}H NMR spectrum. Besides, the integration ratio (I_c/I_g) was extremely close to 2:3, confirming the intact structures of well-defined POSS-(SS-PEG)$_8$ polymer. ^{13}C NMR spectrum also gave the distinct evidence to clarify the rigid framework.

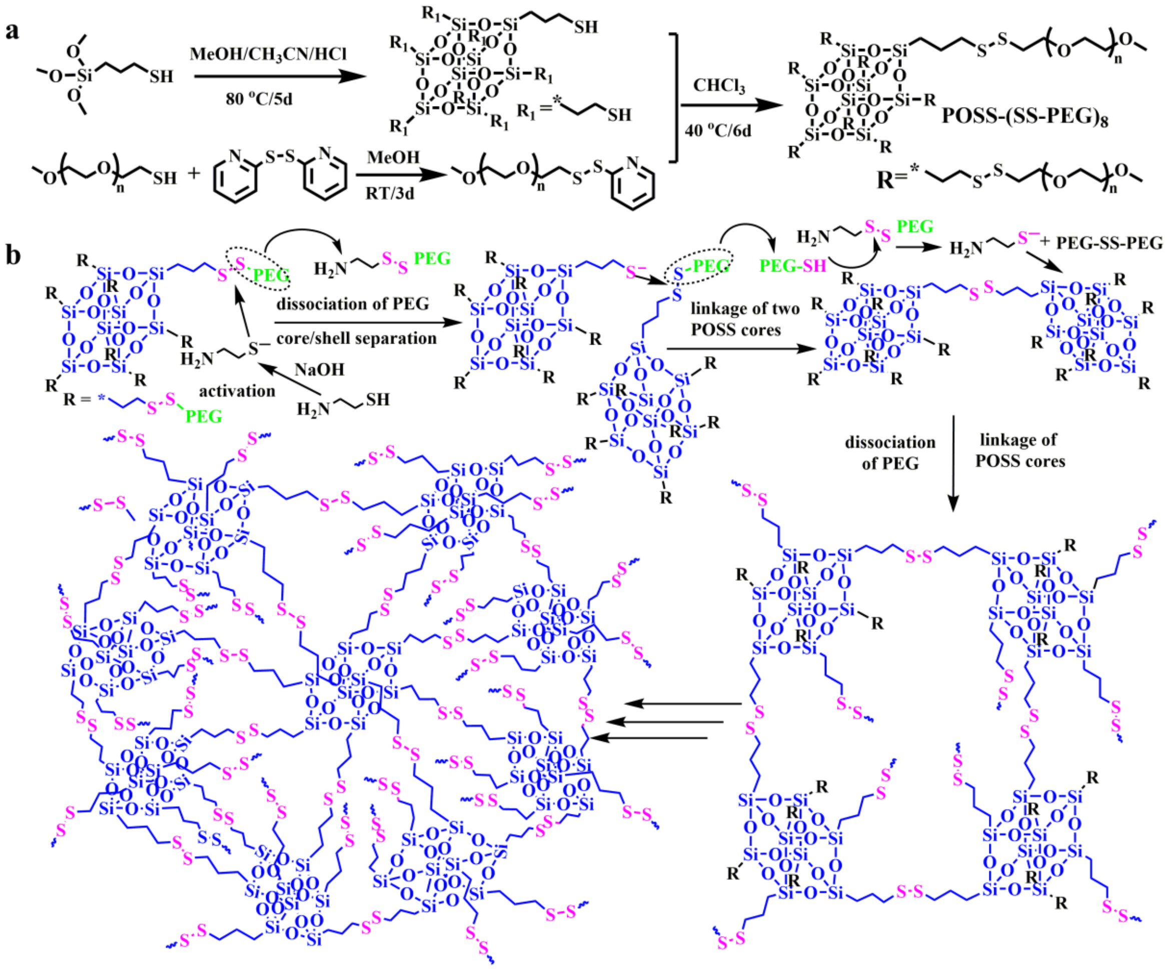

Supplementary Figure 2. Synthesis route and thiol-disulfide exchange reaction mechanism. (**a**) Synthesis route of the POSS-(SS-PEG)$_8$ polymer. The precursor, POSS-(SS-PEG)$_8$, was yielded by modification of POSS-(SH)$_8$ using PEG-SS-2TP. (**b**) The mechanism of pH-triggered thiol-disulfide exchange reaction. Thiolates were initiators of the reaction, and can be produced from deprotonation of thiols in the presence of a small amount of cysteamine in the basified solution. Controlling the solution pH could reversibly deprotonate thiols and protonate thiolates, turning on/off the thiol-disulfide exchange reaction. The exchange reaction resulted in core/shell separation: dissociation of the PEG shells and cross-linking of the POSS cores.

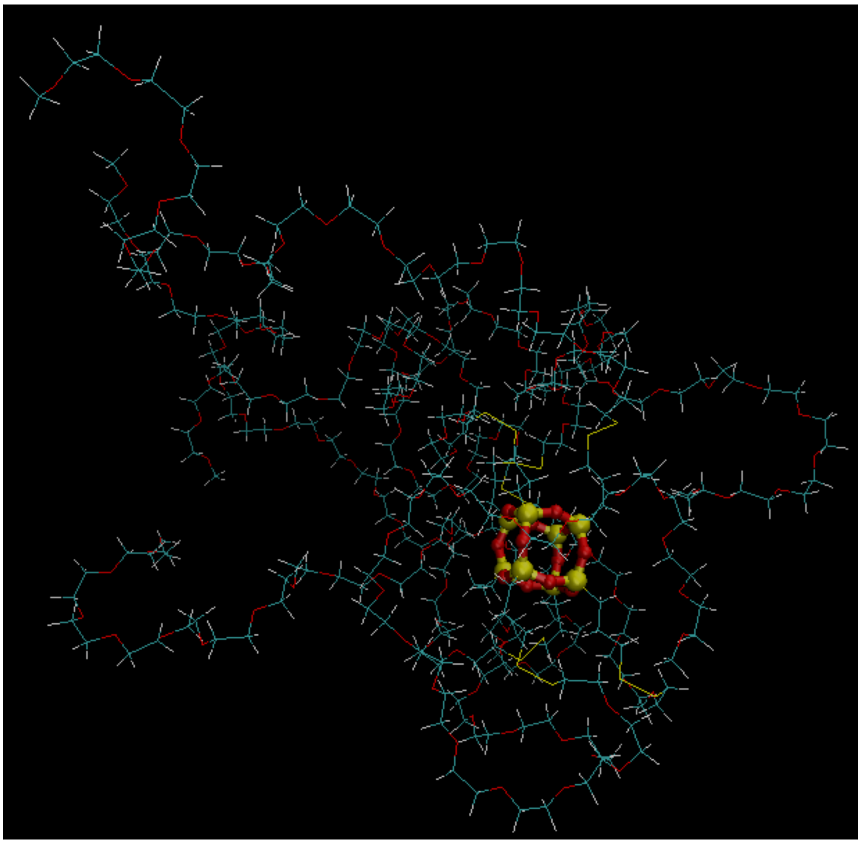

Supplementary Figure 3. MD simulation. After the computational simulation, the size of POSS-(SS-PEG)$_8$ was about 3 nm. The inner cube represents the POSS core, wherein the yellow cylinder represents the silicon (Si) atom and red cylinder represents the oxygen (O) atom. The outer linkage represents the PEG shell, wherein the red cylinder represents the oxygen (O) atom, yellow cylinder represents the sulphur (S) atom, wathet blue cylinder represents the carbon (C) atom and white cylinder represents the hydrogen (H) atom.

Supplementary Table 1. Box size of simulation systems.

Degree of polymerization	X (nm)	Y (nm)	Z (nm)
1	8.55	8.55	8.55
2	8.94	8.94	8.94
4	12.58	12.58	12.58
6	14.46	14.46	18.47

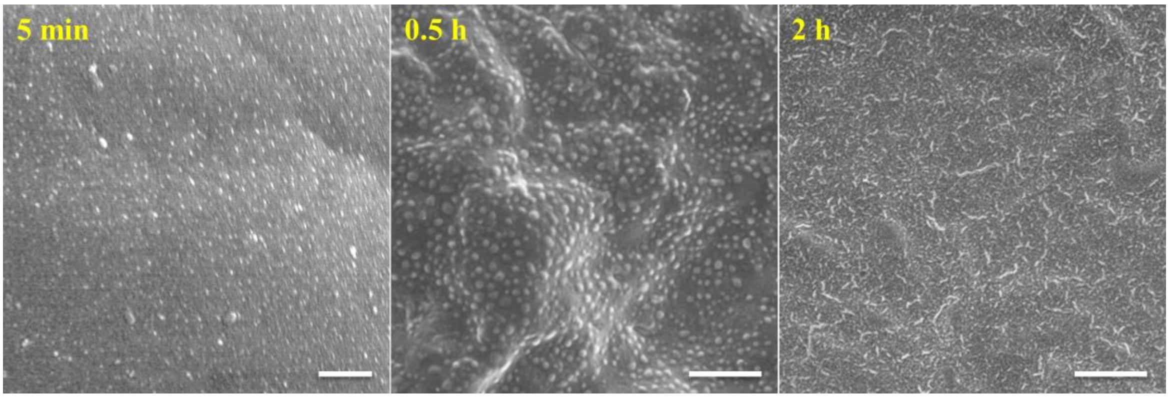

Supplementary Figure 4. Hierarchically axial growth of the micelles. After neutralizing the aqueous solution and removing the by-products before dialysis, all the samples were prepared from the neutralized aqueous solution unless otherwise noted. SEM images showing self-assembled evolution of POSS-SS-$(PEG)_8$ with the concentration of 1 mg/mL in pH 8 aqueous solutions for various times. Scale bars: 0.5 μm.

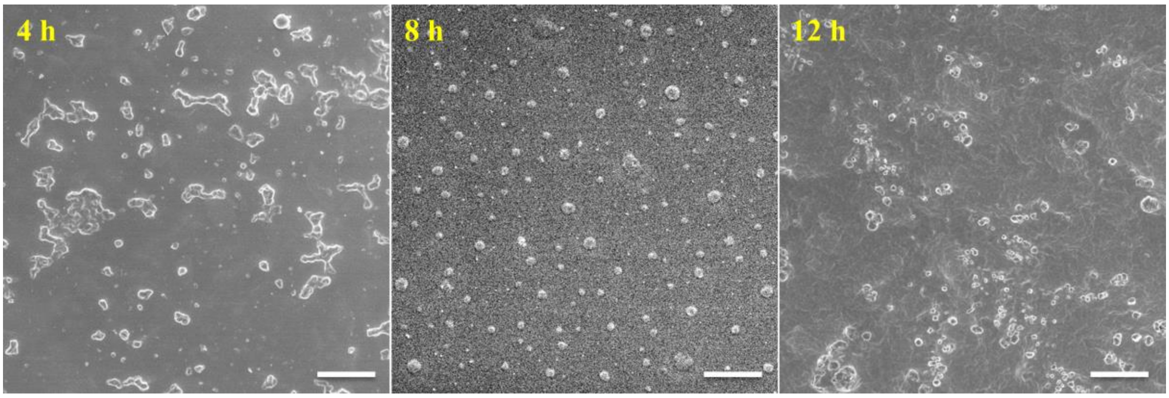

Supplementary Figure 5. Hierarchical bending, cyclization and radial connection of the aggregates. After neutralizing the aqueous solution and removing the by-products before dialysis, all the samples were prepared from the neutralized aqueous solution unless otherwise noted. SEM images showing self-assembled evolution of POSS-SS-$(PEG)_8$ with the concentration of 1 mg/mL in pH 8 solutions for various times. Scale bars: 0.5 μm.

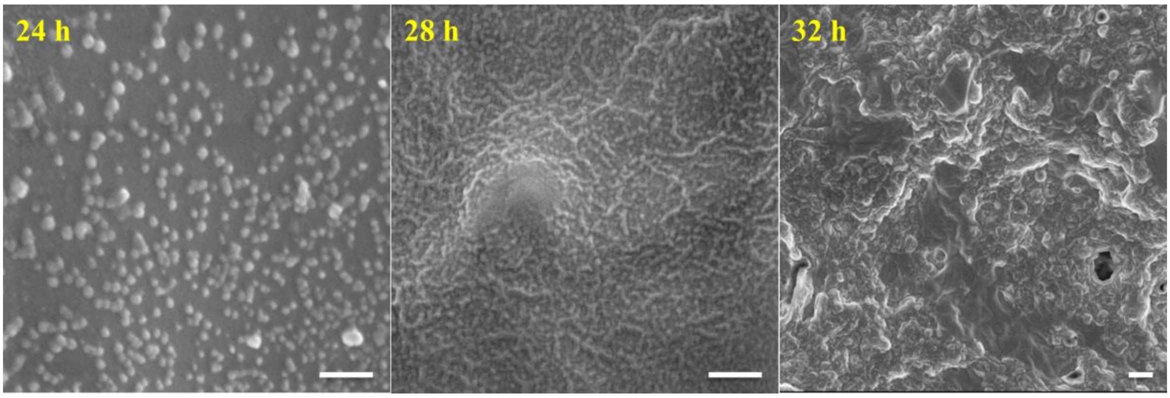

Supplementary Figure 6. Hierarchically nanostructural orientation growth at micro- and macro-scales. After neutralizing the aqueous solution and removing the by-products before dialysis, all the samples were prepared from the neutralized aqueous solution unless otherwise noted. SEM images showing self-assembled evolution of POSS-SS-$(PEG)_8$ with the concentration of 1 mg/mL in pH 8 solutions for various times. Scale bars: 0.5 μm.

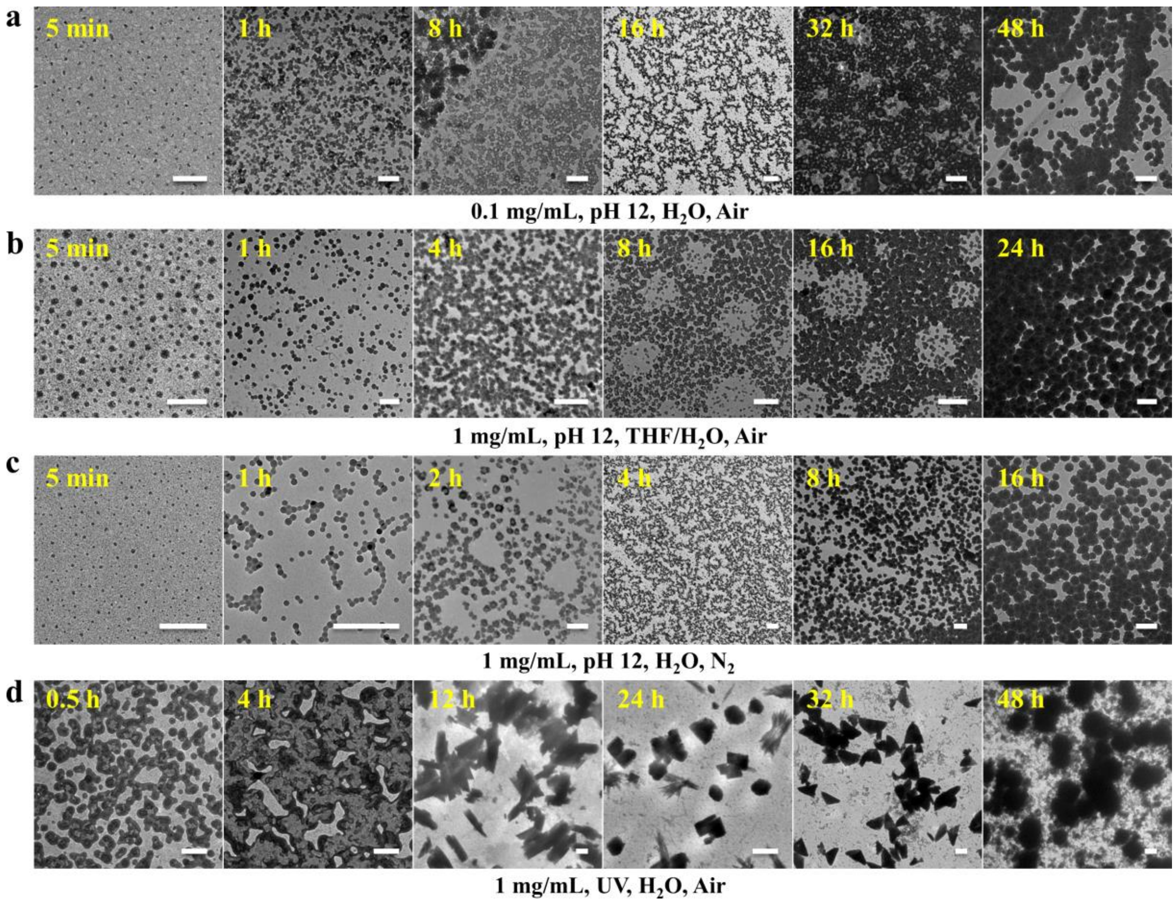

Supplementary Figure 7. Investigation on thiol-disulfide exchange reaction and assembly conditions for the self-assembled evolution. After neutralizing the aqueous solutions and removing the by-products before dialysis, all the samples were prepared from the neutralized aqueous solution unless otherwise noted. TEM images showing the self-assembled evolution of POSS-SS-$(PEG)_8$ with various conditions with (**a**) 0.1 mg/mL in pH 12 aqueous solutions at air atmosphere for various times; (**b**) 1 mg/mL in THF/H_2O (v/v = 1:4, pH 12) solutions at air atmosphere for various times; (**c**) 1 mg/mL in pH 12 aqueous solutions at N_2 atmosphere for various times and (**d**) 1 mg/mL in neutral aqueous solutions under UV irradiation at air atmosphere for various times.

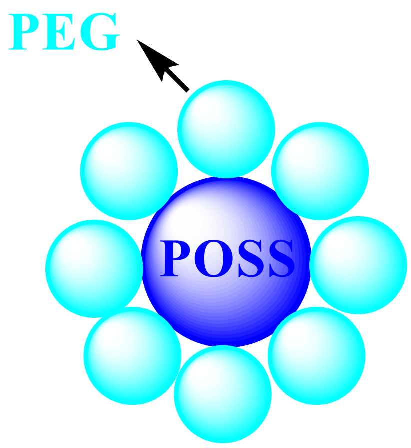

Supplementary Figure 8. CG model of DPD simulation. Scheme of a single model of POSS-(SS-PEG)$_8$ molecule. A blue sphere represents one POSS core and cyan spheres represent the PEG shells.

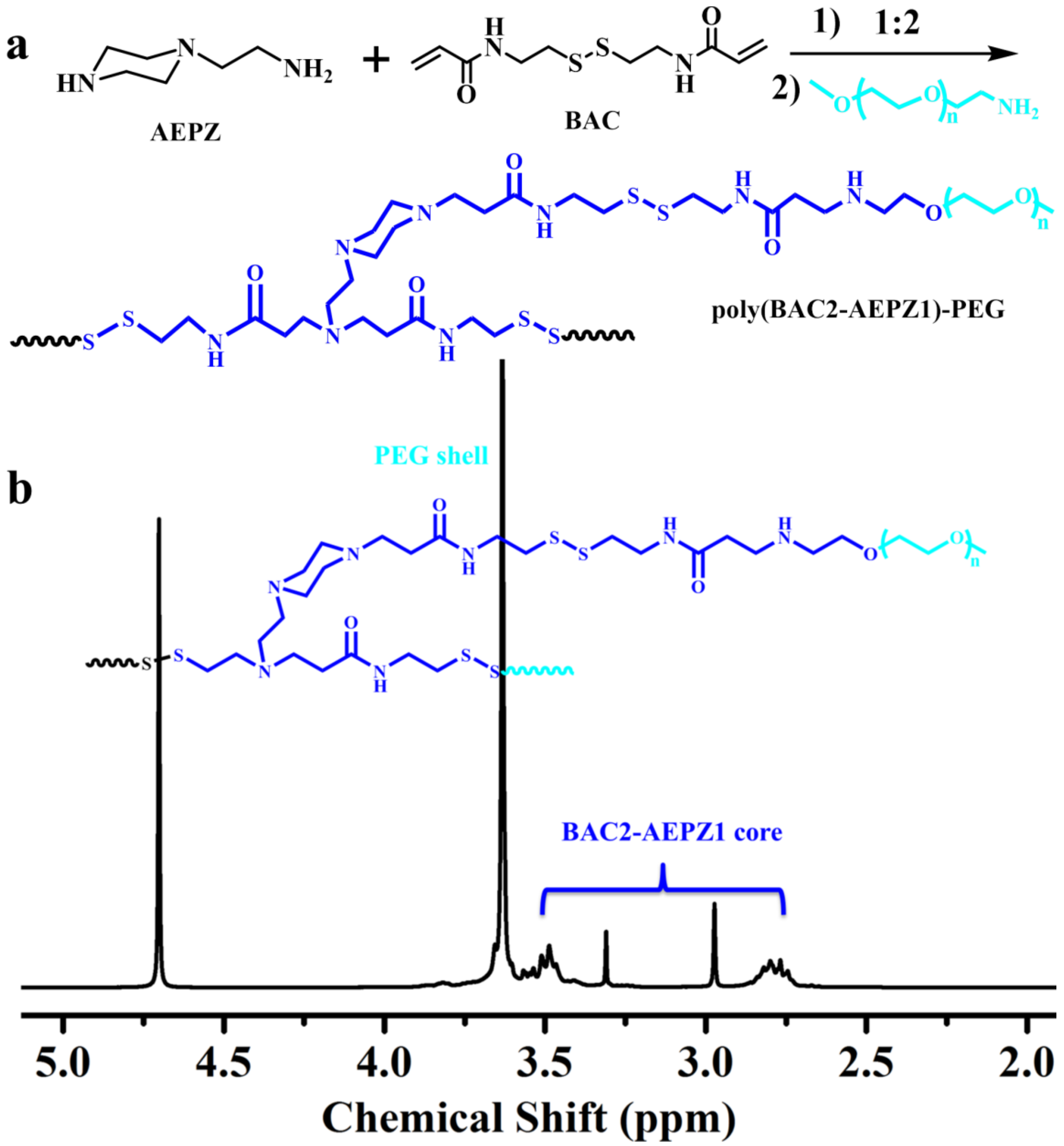

Supplementary Figure 9. A control sample of disulfide-linked hyperbranched polymer with a random core. (**a**) Synthesis of the poly(BAC2-AEPZ1)-PEG polymer. The precursor with terminal vinyl groups was first produced by the reaction of an AEPZ and a double-molar BAC. Then the vinyl groups were sealed by NH_2-PEG to yield a core-shell hyperbranched polymer, wherein the dendritic cores were soft and heterogeneous. (**b**) ^{1}H NMR spectrum of poly(BAC2-AEPZ1)-PEG polymer at D_2O. The resonance peaks located at δ 2.62-3.56 ppm were assigned to the BAC2-AEPZ1 core while the signals at δ 3.3 and 3.5-4.1 ppm were assigned to the PEG chain in the ^{1}H NMR spectrum.

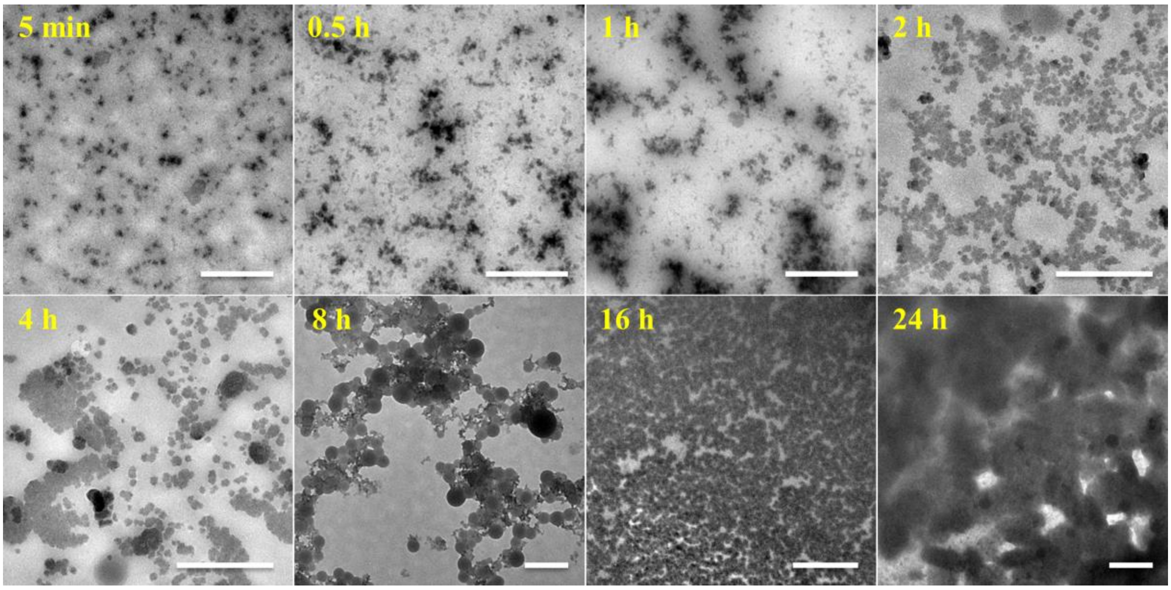

Supplementary Figure 10. Hierarchical self-assembly behavior of the poly(BAC2-AEPZ1)-PEG in alkaline solutions. After neutralizing the aqueous solution and removing the by-products before dialysis, all the samples were prepared from the neutralized aqueous solution unless otherwise noted. TEM images showing self-assembled evolution of poly(BAC2-AEPZ1)-PEG with the concentration of 1 mg/mL in pH 12 aqueous solutions for various times. Scale bars: 0.5 μm. In this process, the morphology evolution was grown toward the axial direction at first on account of the steric hindrance areas from hydrophobic domains and the swollen layers from stretched PEG shells at the middle of micelles. Along with the gradual formation of irregular links among the cores, the superiority on the axial growth was impaired, resulting in the random arrangement and accumulation of the solid nanoparticles. However, the elliptic morphology was finally produced, which also indicated the true hydrogel formation process from the nanoscopic solution aggregates to the macroscopic hydrogel particles in the aqueous solutions.

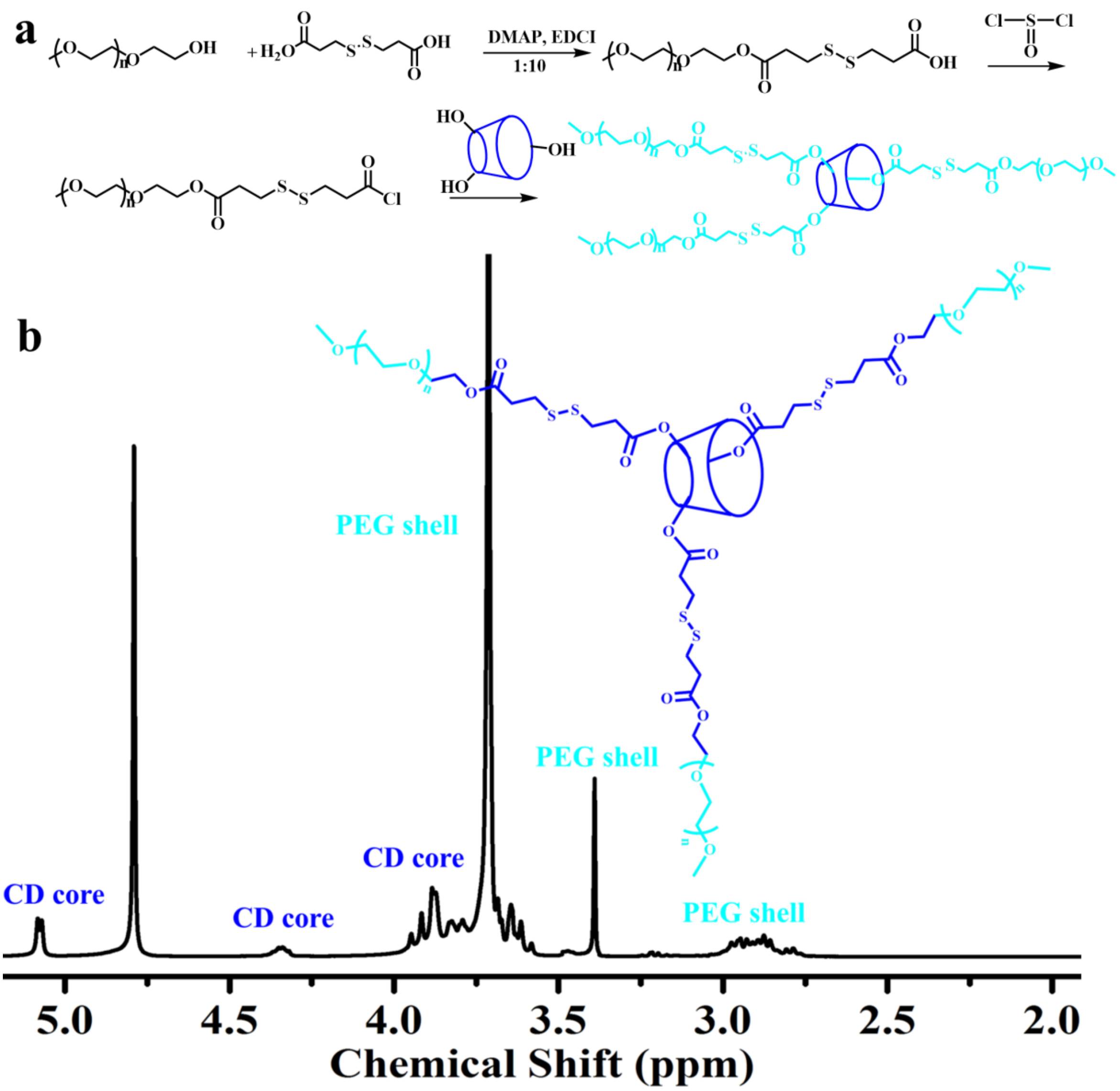

Supplementary Figure 11. A control sample of disulfide-linked star-shaped polymer with an asymmetric core. (**a**) Synthesis of the CD-(SS-PEG)$_{3-4}$ polymer. The precursor, CD-(SS-PEG)$_{3-4}$, was yielded using a well-defined β-CD core and activated PEG-SS-COCl by acylation reaction. (**b**) ^{1}H NMR spectrum of CD-(SS-PEG)$_{3-4}$ polymer at D_2O. The resonance peaks located at δ 3.88, 4.36 and 5.15 ppm were assigned to β-CD moieties while the signals at δ 3.3 and 3.5-4.1 ppm were assigned to the PEG chain in the ^{1}H NMR spectrum. According the typical integration ratio, the average grafting extent of PEG onto the CD was about 3.6.

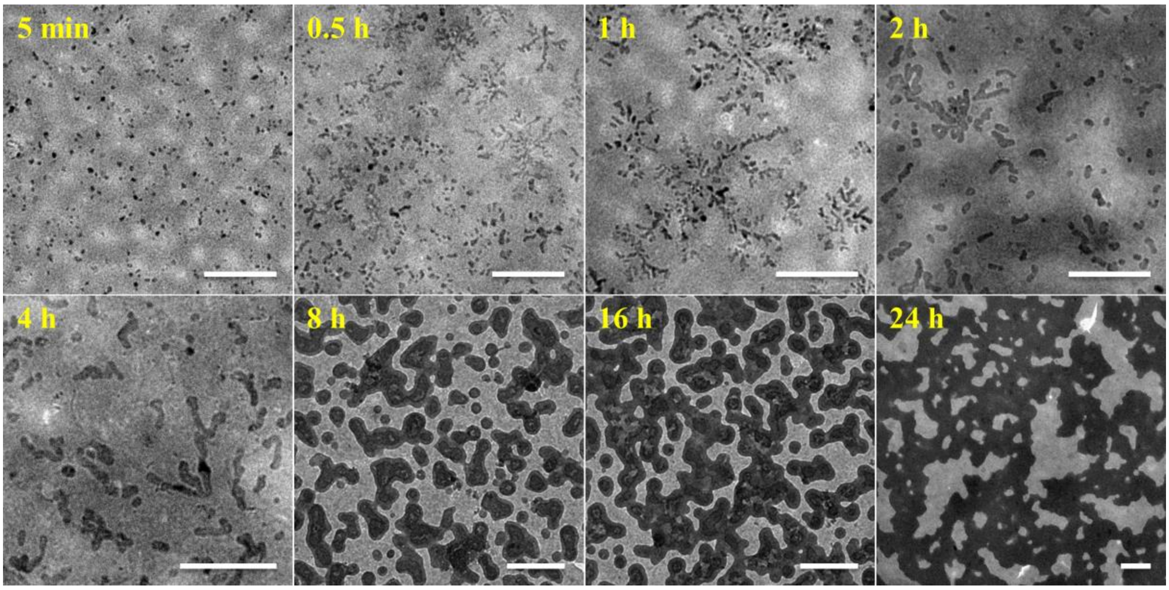

Supplementary Figure 12. Hierarchical self-assembly behavior of the *β*-CD-(SS-PEG)$_{3-4}$ in alkaline solutions. After neutralizing the aqueous solution and removing the by-products before dialysis, all the samples were prepared from the neutralized aqueous solution unless otherwise noted. TEM images showing self-assembled evolution of *β*-CD-(SS-PEG)$_{3-4}$ with the concentration of 1 mg/mL in pH 12 aqueous solutions for various times. Scale bars: 0.5 µm. In this process, the morphology evolution was grown toward the axial and dendritic direction on account of the steric hindrance areas from CD-embedded domains and the swollen layers from stretched PEG shells at the middle of micelles, presenting the unequivocal step-change shifts in cylindrical micelle length and thickness. However, the *β*-CD-(SS-PEG)$_{3-4}$ polymer cannot achieve the hierarchical cyclization and radial crosslinking behaviors on account of the asymmetrical cone, ring tensions and huge steric hindrance of the cyclodextrin, which further suggested the siginficance of symmetry for the inner core for the sequential morphlogy transition.

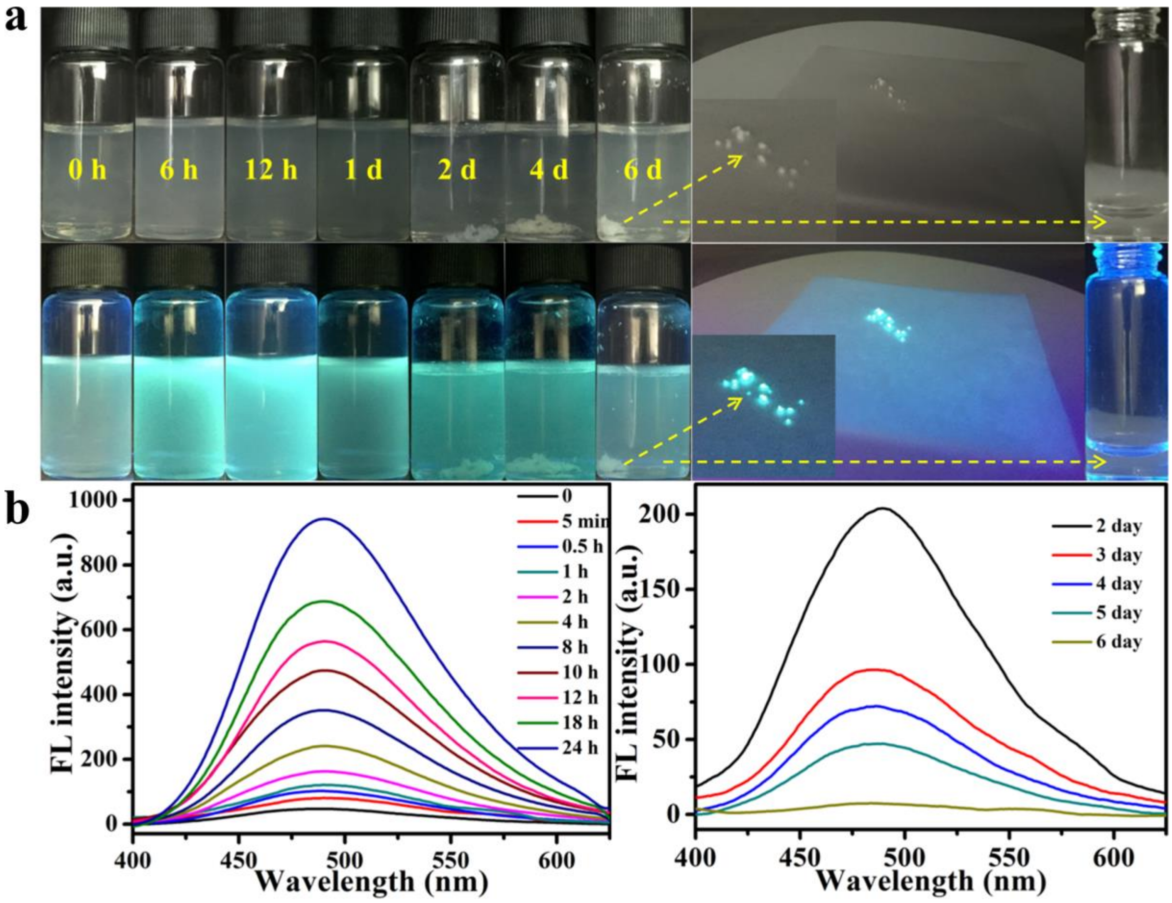

Supplementary Figure 13. Quantitative measurement of the loading efficiency. (**a**) Photographs and (**b**) fluorescence spectra of POSS-(SS-PEG)$_8$@TPE-CHO aggregates in pH 12 THF/H_2O mixtures as a function of time. When the TPE-CHO molecule (1 mg) was added into the THF/H_2O (v/v = 1:4) mixtures of POSS-(SS-PEG)$_8$ polymer (5 mg), the solution emitted weak fluorescence due to that the TPE-CHO had not efficient capacity to achieve the critical aggregate state of AIE effects. Along with the activation of morphology evolution, more and more hydrophobic molecules were gradually encapsulated and aggregated in the self-assemblies originating from the strong hydrophobic interactions, leading to the growth of fluorescent intensity. After sufficient incubation time, the hydrophobicity of aggregates was intensely enhanced so that the precipitates occurred and gradually increased at the bottom, which emitted strong cyan fluorescent lights while the supernatant liquid was nearly non-emissive, suggesting the complete encapsulation of TPE-CHO molecules into the polymeric assemblies.

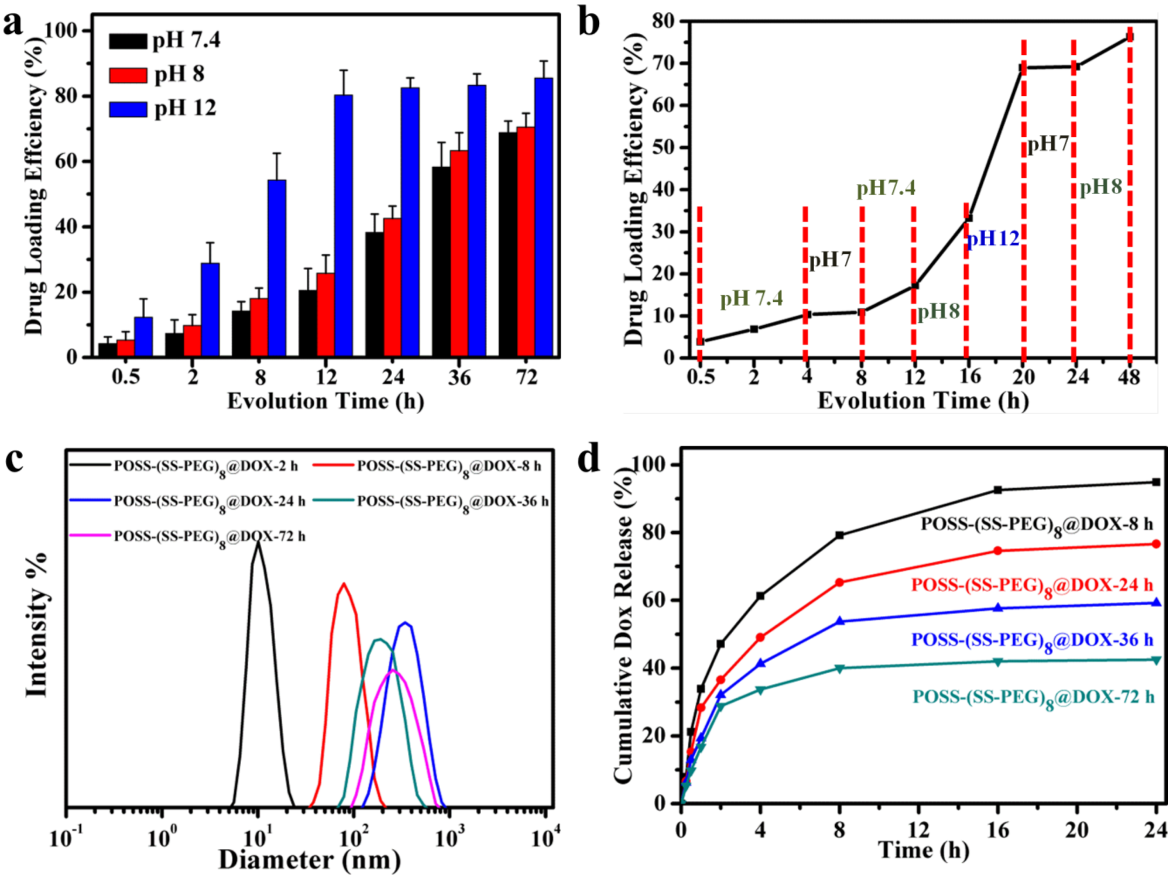

Supplementary Figure 14. Loading and *in vitro* release of DOX. (**a**) DOX-loaded efficiency of the POSS-(SS-PEG)$_8$ polymer in various pH (7.4, 8 and 12) mixtures (THF/H_2O) as a function of time (n = 6 per group). All the data were presented as the average ± standard deviation. (**b**) DOX-loaded efficiency change of the POSS-(SS-PEG)$_8$ polymer in mixtures (THF/H_2O) with activation, acceleration, termination and reactivation functions under the control of system pH (7, 7.4, 8 and 12) as a function of time. The red dashed line represents the specified time interval. For example, strong alkaline environment accelerated the drug loading into evolved aggregates while neutral solution basically suspended drug encapsulation, thereby achieving the controlled drug loading behavior only by manipulation of solution pH value. (**c**) Size change of POSS-(SS-PEG)$_8$@DOX aggregates in pH 7.4 solutions for various thiol-disulfide exchange reaction times determined by DLS measurement. Along with the gradual encapsulation of DOX molecules into the micelles, the size increased from 15 to 400 nm

within 24 h. However, the size of DOX-loaded micelles was reduced into ~170 nm after 36 h on account of the dissociation of numerous PEG shells that severely decreased the hydrophilicity of aggregates. (**d**) DTT-triggered release of DOX from POSS-(SS-PEG)$_8$@DOX aggregates with various thiol-disulfide exchange reaction times as a function of time. Since the compaction degree of drug-loaded micelles was relevant to DTT permeation capacity, these redox-responsive aggregates containing various drug loading contents presented the distinct DOX release process in the presence of 10 mM DTT at pH 7.4 PBS. The fast release of DOX was attributed to the sequential swelling, cracking and degradation of polymeric micelles in the first 4 h, but the compacter hydrophobic areas were difficult to be fully disrupted and disassembled at 37 °C.

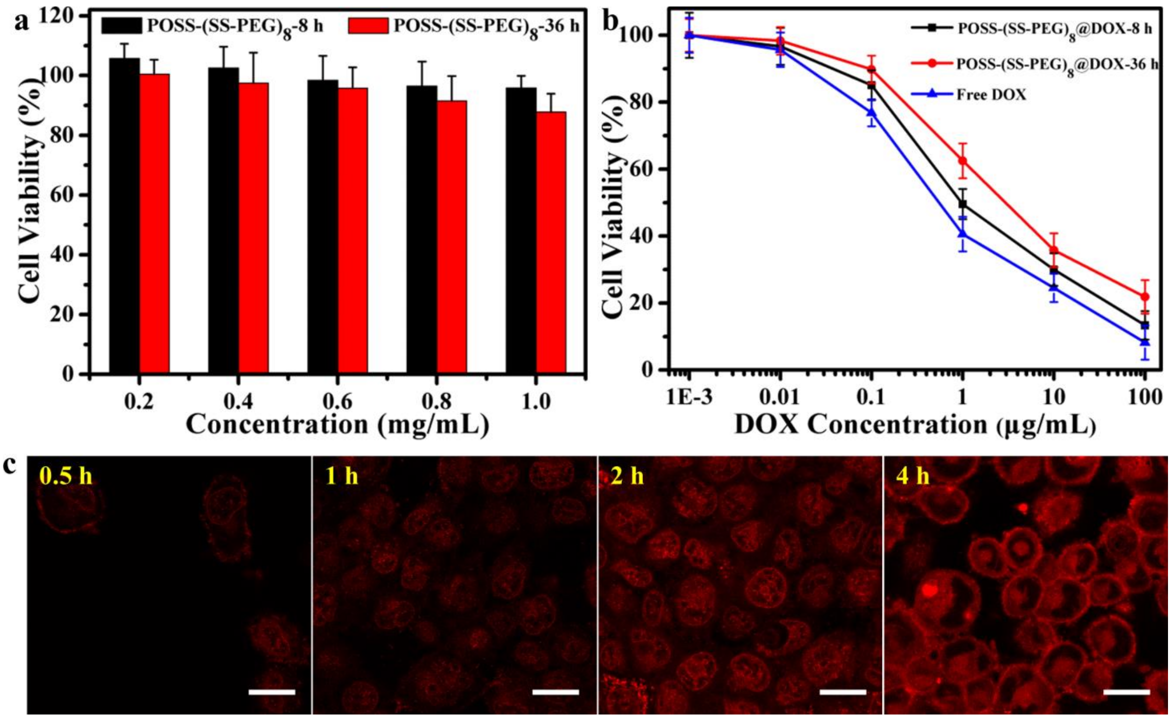

Supplementary Figure 15. Cellular uptake and intracellular drug release in drug delivery system. (**a**) Cytotoxicity of POSS-(SS-PEG)$_8$ nanoparticles (exchange reaction at pH 7.4 for 8 h and 36 h) to the MCF-7 cells (n = 5 per group) following 24 h of incubation. All the data were presented as the average ± standard deviation. Two micelles were nontoxic to MCF-7 cells due to their quick degradation capacity in the presence of high levels of reductive agents. The cell viability was more than 90% until a tested concentration of 1.0 mg/mL, demonstrating the excellent biocompatibility of biodegradable micelles. (**b**) Viabilities of the MCF-7 cells (n = 5 per group) following 24 h of incubation with POSS-(SS-PEG)$_8$@DOX aggregates (exchange reaction at pH 7.4 for 8 h and 36 h) and free DOX as a function of DOX dosages. All the data were presented as the average ± standard deviation. These two DOX-loaded micelles had a similar toxicity as free DOX in a low concentration. In a high concentration, DOX molecules can diffuse into cells rapidly, whereas the assembled micelles had to be endocytosed to enter the cells, which made free DOX molecules quicker than DOX-loaded aggregates in internalization, thus presenting high efficiency in cancer cell inhibition. (**c**) Confocal laser scanning

microscopy images of the MCF-7 cells after incubation with POSS-(SS-PEG)$_8$@DOX-8 h aggregates (exchange reaction at pH 7.4 for various times with the excitation of 488 nm for DOX. Scale bars: 20 μm. The red fluorescence was directly observed within the MCF-7 cells in the first 0.5 h, suggesting that these DOX-loaded aggregates had been successfully internalized by tumor cells with rapid DOX release from the aggregates and the localization in the endosome. Along with the prolonged incubation time for 4 h, the intracellular fluorescent intensity tended to be stronger within the cell nucleus, indicating the efficient release of DOX from the nanoparticles and their further escape from the endosome/lysosome to the nucleus.
